# Electron transport chain activity is a predictor and target for venetoclax sensitivity in multiple myeloma

**DOI:** 10.1038/s41467-020-15051-z

**Published:** 2020-03-06

**Authors:** Richa Bajpai, Aditi Sharma, Abhinav Achreja, Claudia L. Edgar, Changyong Wei, Arusha A. Siddiqa, Vikas A. Gupta, Shannon M. Matulis, Samuel K. McBrayer, Anjali Mittal, Manali Rupji, Benjamin G. Barwick, Sagar Lonial, Ajay K. Nooka, Lawrence H. Boise, Deepak Nagrath, Mala Shanmugam

**Affiliations:** 1 Department of Hematology and Medical Oncology, Winship Cancer Institute, School of Medicine, Emory University, Atlanta, GA USA; 2 Department of Biomedical Engineering, University of Michigan, Ann Arbor, MI USA; 3 Biointerfaces Institute, University of Michigan, Ann Arbor, MI USA; 4 Children’s Medical Center Research Institute, University of Texas Southwestern Medical Center, Dallas, TX USA; 5 Department of Chemical Engineering, University of Michigan, Ann Arbor, MI USA; 6 Department of Biostatistics and Bioinformatics Shared Resource, Winship Cancer Institute, Emory University, Atlanta, GA USA

**Keywords:** Enzymes, Myeloma, Cell biology, Molecular biology, Cancer therapy

## Abstract

The BCL-2 antagonist venetoclax is highly effective in multiple myeloma (MM) patients exhibiting the *11;14* translocation, the mechanistic basis of which is unknown. In evaluating cellular energetics and metabolism of t(11;14) and non-t(11;14) MM, we determine that venetoclax-sensitive myeloma has reduced mitochondrial respiration. Consistent with this, low electron transport chain (ETC) Complex I and Complex II activities correlate with venetoclax sensitivity. Inhibition of Complex I, using IACS-010759, an orally bioavailable Complex I inhibitor in clinical trials, as well as succinate ubiquinone reductase (SQR) activity of Complex II, using thenoyltrifluoroacetone (TTFA) or introduction of SDHC R72C mutant, independently sensitize resistant MM to venetoclax. We demonstrate that ETC inhibition increases BCL-2 dependence and the ‘primed’ state via the ATF4-BIM/NOXA axis. Further, SQR activity correlates with venetoclax sensitivity in patient samples irrespective of t(11;14) status. Use of SQR activity in a functional-biomarker informed manner may better select for MM patients responsive to venetoclax therapy.

## Abstract (web summary)

Venetoclax monotherapy is effective in 40% of t(11:14) positive multiple myeloma (MM). Here, the authors show that electron transport chain complex I (CI) and complex II (CII) activity predict MM sensitivity to venetoclax, and inhibition of CI with IACS-010759 or CII with TTFA increase sensitivity.

## Introduction

Multiple myeloma (MM) is an incurable plasma cell malignancy. MM accounted for approximately 12,590 deaths and 30,280 new diagnoses in the US, in 2017^1,2^. Despite the administration of next-generation proteasome inhibitors, autologous transplant and immunomodulatory compounds in steroid-supplemented combinatorial strategies, most patients succumb to disease^2^. Myeloma comprises a genetically complex multiclonal cancer. Relapse and the development of refractory disease occur primarily due to the emergence of subclonal resistant populations of cells and the inability to induce cell death in the residual population. Identifying ways to engage common distal effectors of apoptosis can circumvent the proximal resistance promoting mechanisms that develop.

The BCL-2 family of proteins is central to regulating the intrinsic pathway of programmed cell death, and novel approaches to targeting these proteins continue to demonstrate therapeutic promise. The BCL-2 proteins are divided into four groups based on structure and function. The antiapoptotic BCL-2, BCL-xL, MCL-1, BCL-w and BFL-1 proteins prevent death by sequestering proapoptotic BH3-only activator proteins BIM, PUMA and BID. The proapoptotic BH3-only sensitizer proteins, NOXA, BAD, BMF and BIK, release activator proapoptotics by binding antiapoptotic proteins. These released proapoptotics activate effector proteins BAX and/or BAK, which oligomerize and permeabilize the outer mitochondrial membrane releasing cytochrome c to activate subsequent steps of apoptosis^3,4^. The “dependency” on a particular antiapoptotic protein refers to the antiapoptotic that is most pertinent for maintaining a cell’s survival, which is in turn dictated by the sufficiency of proapoptotics bound to that antiapoptotic which upon release would commit a cell to apoptosis^5^. The BH3 activator, BIM, is highly expressed in cells of hematopoietic origin^6^ and evaluation of BH3 activators bound to antiapoptotics in MM demonstrated that BIM is the most relevant BH3 activator bound to BCL-2^7^. Despite heavy reliance of MM on MCL-1^8,9^ and correlation of MCL-1 levels with poor prognoses^10^, targeting BIM−BCL-2 interactions has been shown to induce apoptosis even in MCL-1-dependent MM^7^.

BH3 mimetics are a class of small molecules that block the interaction of specific proapoptotics with cognate antiapoptotics, releasing bound proapoptotic activators^4^. Venetoclax is one such selective, potent BCL-2 antagonist^11^. It is highly effective in BCL-2-dependent malignancies and FDA-approved for the treatment of chronic lymphocytic leukemia (CLL)^12^ and with hypomethylating agents azacitidine or decitabine (NCT02203773)^13^ or low dose cytarabine (NCT02287233) in acute myeloid leukemia (AML). Intriguingly, a small fraction (approximately 7%) of MM patients (about 40% of the 15–20% of patients exhibiting the *11;14* translocation) respond to single-agent venetoclax^14–16^. Given the plethora of new myeloma therapies, there is need for precision therapy informed by biomarkers or molecular traits. Understanding the basis for single-agent efficacy of venetoclax in t(11;14) myeloma can be highly informative for identifying patients who will benefit from single-agent venetoclax therapy as well as identify targets for developing rational venetoclax containing combinations to expand use of venetoclax beyond the small cohort of patients currently sensitive to venetoclax monotherapy.

Metabolites regulate the primed state, i.e. proximity to the apoptotic threshold, by regulating the expression and binding properties of pro- and antiapoptotic BCL-2 family members^17–21^. Metabolites such as glucose, glutamine and (R)-2HG^22^ have previously been shown to regulate BCL-2, MCL-1, BCL-xL, PUMA, NOXA and BIM expression and/or their interactions. Therefore, it is not surprising that perturbing metabolism can alter dependence and sensitivity to specific BH3 mimetics. We previously reported that glutamine deprivation increases BIM binding to BCL-2 thereby sensitizing MM to venetoclax while supplementation with α-ketoglutarate reversed this sensitivity^23^, affirming metabolic regulation of BCL-2 dependence. We therefore explore the presence of a metabolic basis for t(11;14) myeloma sensitivity to single-agent venetoclax that could aid in identifying (1) venetoclax-sensitive MM in the broader MM population, and (2) metabolic targets that could be inhibited to sensitize resistant MM to venetoclax. Our studies reveal Complex I and the succinate ubiquinone reductase (SQR) activity of Complex II of the ETC as targets for venetoclax sensitization and SQR activity as an assessable predictor of patient response.

## Results

### Venetoclax-sensitive MM exhibits reduced cellular energetics in contrast to venetoclax-resistant MM

**Fig. 1:**
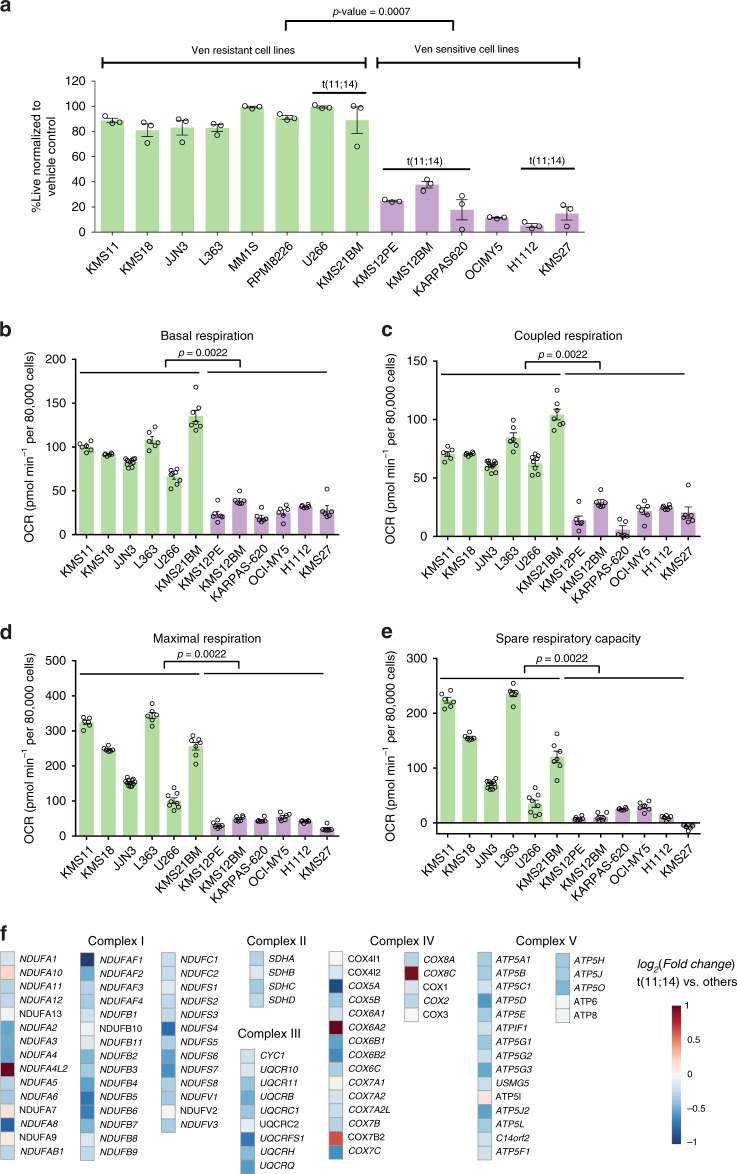
Venetoclax-sensitive MM exhibits reduced cellular energetics in contrast to the venetoclax-resistant cells. **a** MM cell lines treated ±0.5 μM venetoclax (Ven) for 24 h were assessed for cell death by AnnexinV/4′,6-diamidino-2-phenylindole (DAPI) flow cytometric staining. Percent live normalized to vehicle control, with cell lines grouped by sensitivity. *n* = 3 independent experiments. Data are presented as mean values ± SEM. *p* value is calculated using a two-tailed Mann−Whitney test. **b**−**d** MM lines were evaluated for basal, coupled and maximal respiration in a mito stress assay using a Seahorse XF_e_96 analyzer. *n* = 6 replicate Seahorse wells for each cell line except JJN3 (*n* = 11), U266 (*n* = 8) and KMS21BM (*n* = 7). OCR displayed are normalized to live cell number. Data are presented as mean values ± SEM. Venetoclax-sensitive MM exhibited significantly lower basal, maximal and coupled OCR (*p* values = 0.0022) determined after the addition of oligomycin, FCCP and antimycin/rotenone. *p* values are calculated using a two-tailed Mann−Whitney test. **e** Spare respiratory capacity in venetoclax-sensitive and -resistant cells was determined by subtracting basal OCR from maximal OCR. Data are presented as mean values ± SEM. Venetoclax-resistant lines have been shown in green and venetoclax-sensitive lines have been shown in purple bars in (**a**−**e**). **f** Heat map of electron transport chain-specific gene expression in t(11;14) vs. non-t(11;14) patients derived from the CoMMpass trial RNAseq. Statistical significance (adjusted *p* value < 0.01) is highlighted for gene names in bold-italic font*.* Source data are provided as a source data file.

We first assessed differential venetoclax sensitivity in a panel of MM lines. As previously reported^14^, venetoclax elicited significant cytotoxicity primarily in t(11;14) lines (Fig. 1a). The pattern of increased sensitivity of these cell lines was selective for venetoclax and not detected with other standard myeloma therapeutics i.e. bortezomib and melphalan (Supplementary Fig. 1a, b) or sensitivity to the MCL-1 inhibitor S63845 as reported for the KMS12BM, KMS12PE, KMS11, MM.1S, JJN3, RPMI-8226 and L363 lines^24^.

To investigate differential energy metabolism in venetoclax-sensitive and -resistant cells, we performed glucose and glutamine carbon isotope tracing using labeled U^13^C-glucose or U^13^C-glutamine in resistant non-t(11;14) KMS11 and t(11;14) U266, as well as sensitive t(11;14) KMS12PE and non-t(11;14) OCI-MY5 cell lines (Supplementary Fig. 2). We detected lower TCA cycle metabolite levels in the sensitive compared to venetoclax-resistant lines (Supplementary Fig. 2c, f). Glucose-derived carbon contribution to the TCA cycle intermediates was reduced in venetoclax-sensitive cells, reflected in the diminished pool of citrate, succinate, fumarate and malate along with decrease in ^13^C enrichment of citrate, α-ketoglutarate, succinate, fumarate and malate in cells supplemented with U-^13^C-glucose (Supplementary Fig. 2a, d). In contrast, we detected comparable glutamine-derived carbon utilization (Supplementary Fig. 2b, e). The results cannot be explained by reduced nutrient uptake or reduced mitochondrial content in the venetoclax-sensitive lines as these cells do not exhibit a uniformly reduced pattern of glucose or glutamine consumption or proliferation rate (Supplementary Fig. 3a−c), and mitochondrial mass (Supplementary Fig. 4) as assessed by cardiolipin staining^25^.

Next, we evaluated oxygen consumption rates (OCR) in the MM cell lines. Basal, maximal, coupled OCR and spare respiratory capacity (SRC) were found to be significantly lower in all of the sensitive MM lines (Fig. 1b−e). Interestingly, insensitive t(11;14) lines such as U266 and KMS21BM displayed high basal, maximal and coupled respiration while the sensitive line, OCI-MY5, which does not have a 11;14 translocation, exhibited lower respiratory parameters, suggesting that oxygen consumption and potentially electron transport chain (ETC) activity could segregate venetoclax-sensitive and -resistant MM cells. Importantly, SRC, which is the difference in basal and maximal respiration and reflects the potential to elevate cellular bioenergetics and ATP synthesis during oxidative stress or upon partial Complex I inhibition^26^, is also reduced in the sensitive cells. In sum, these results suggest that the ETC activities may be suppressed in venetoclax-sensitive cells.

To discern the basis for differential OCR, we next queried the CoMMpass MM trial (NCT0145429, IA11) RNAseq data, to examine ETC gene expression. Since we do not know the response of these patients to venetoclax, we used t(11;14) as proxy for venetoclax sensitivity. Interestingly, this analysis demonstrated marked suppression of ETC-related genes in patients with t(11;14) MM (Fig. 1f) which is in line with our OCR assessment of t(11;14) and venetoclax-sensitive cell lines (Fig. 1).

### Venetoclax-sensitive MM exhibits reduced Complex I and Complex II activity

Given reduced OCR in sensitive cells, we examined the activities of mitochondrial Complex I and II. Mitochondrial Complex I and II receive electrons from the electron donors NADH or FADH_2_ respectively, that are transferred to the terminal electron acceptor (O_2_) via a series of redox reactions. Complex I or NADH:Coenzyme Q:oxidoreductase is the largest multimeric respiratory complex of ETC comprised of over 40 subunits with its catalytic subunit containing the NADH binding site. Electrons from NADH are transferred to FMN and then to FeS clusters and finally to ubiquinone where ubiquinone is reduced to ubiquinol^27^. Complex II is the only enzyme that has a direct role both in the TCA cycle and ETC. Complex II/Succinate dehydrogenase (SDH) consists of four subunits: SDHA, SDHB, SDHC and SDHD that contain distinct dehydrogenase and oxidoreductase enzymatic activities^28^. SDHA exposed to the mitochondrial matrix contains the catalytic dicarboxylate binding site for succinate. SDHA is responsible for the SDH activity of Complex II and oxidizes succinate to fumarate in addition to reducing FAD to FADH_2_. Electrons from FADH_2_ are then transferred sequentially to Fe-S clusters in SDHB and then to ubiquinone at the Qp site formed by SDHC and D, embedded in the mitochondrial inner membrane^29^. The reduction of ubiquinone bound in the Qp site to ubiquinol is referred to as the succinate ubiquinone reductase (SQR) activity of Complex II.

We measured Complex I activity using an assay that relies on immunocapture of Complex I from freshly prepared cellular protein extracts. The NADH dehydrogenase activity of immunocaptured Complex I was determined by the oxidation of NADH and simultaneous reduction of the provided dye resulting in increase in absorbance. SDH and SQR activities were measured in live permeabilized cells, containing intact mitochondria pretreated with inhibitors of Complex I (rotenone), Complex III (antimycin) and Complex IV (sodium azide), allowing for selective measurement of SQR or SDH activity. In the assay, SDH activity correlates with electron transfer from succinate to the Fe-S clusters to the water-soluble dye 5-methyl-phenazinium methyl sulfate (PMS) to the final acceptor 3-(4,5-dimethylthiazol-2-yl)-2,5-diphenyltetrazolium bromide (MTT). On the other hand, SQR activity correlates with electron transfer from succinate to the Fe-S clusters to decylubiquinone bound at the Qp site and then final transfer to the redox sensitive dye, 2,6-dichlorophenolindophenol (DCPIP)^30^. The assay is validated by detection of SDH activity only upon addition of the electron donor i.e. succinate and inhibition of activity upon addition of malonate (dicarboxylate binding site competitor) or selective SQR inhibition with thenoyltrifluoroacetone (TTFA) as demonstrated in Supplementary Fig. 5.

**Fig. 2:**
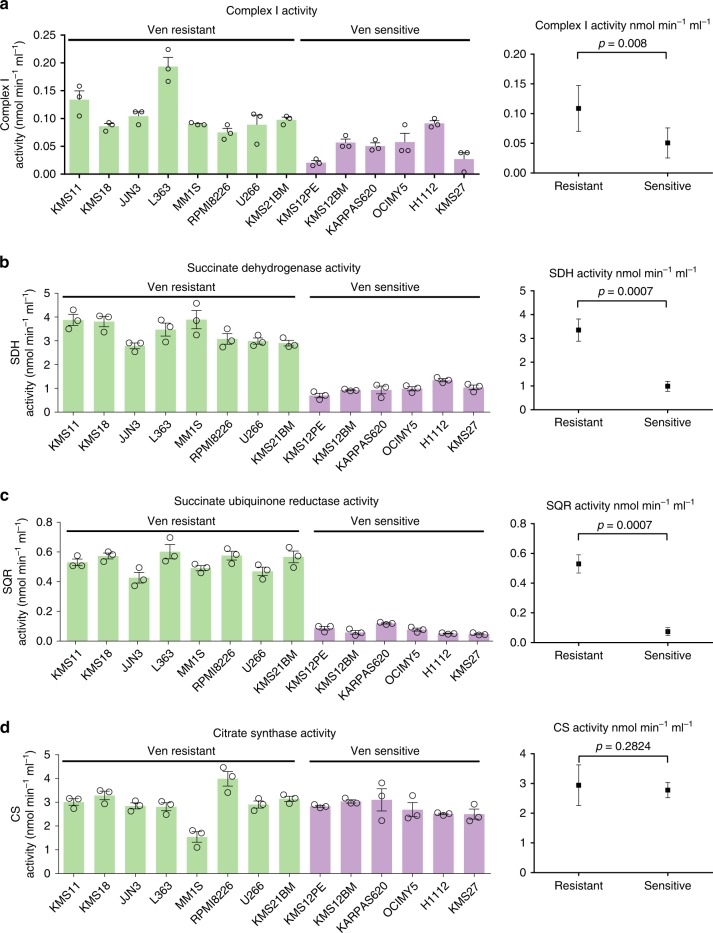
Venetoclax-sensitive MM exhibits reduced Complex I and Complex II activity. **a** Complex I activity was assessed in the indicated lines as described in Materials and Methods. **b** SDH activity and **c** SQR activity were assessed in gently permeabilized whole cells supplemented with succinate and Complex I, III and IV inhibitors. **d** CS activity was assessed as described in Materials and Methods. Complex I, SQR and SDH activity were significantly different in sensitive vs. resistant lines (*p* values 0.008, 0.0007 and 0.0007 respectively) while CS activity did not differ significantly (*p* value = 0.2824*)*. *n* = 3 independent experiments. Data are presented as mean values ± SEM. *p* values are calculated using a two-tailed Mann−Whitney test. Venetoclax-resistant lines have been shown in green and venetoclax-sensitive lines have been shown in purple bars in (**a**−**d**). Source data are provided as a source data file.

We determined that venetoclax-sensitive cells exhibit significantly lower Complex I activity (*p* value = 0.008) and SQR and SDH activities (*p* values = 0.0007) than resistant lines including t(11;14) U266 and KMS21BM (Fig. 2a−c), correlating with venetoclax sensitivity. The activity of citrate synthase (a TCA cycle enzyme) was similar in sensitive and resistant lines (*p* value = 0.2824*)* (Fig. 2d) suggesting the presence of an equivalently functional TCA cycle enzyme activity in contrast to the ETC activities. Interestingly, Complex I subunit (NDUFS2) and SDH subunit protein expression levels (Supplementary Fig. 6a, b) did not correlate with Complex I, SDH or SQR activity (Fig. 2a−c), underscoring the importance of assessing enzymatic activity over subunit gene expression to explain their functional contribution.

We also investigated whether other cancers demonstrate a correlation between low SQR activity and venetoclax sensitivity. We evaluated DLBCL lines ((SUDHL-4, SUDHL-6, U2932, OCI-LY19, OCI-LY10, HBL-1) for both SQR activity and venetoclax sensitivity. As shown in Supplementary Fig. 7, we did not detect a correlation between low SQR activity and venetoclax sensitivity suggesting that the relationship between low SQR activity and BCL-2 dependence may be plasma cell context specific (further elaborated upon in Discussion).

### Inhibition of SQR with Qp site inhibitor TTFA sensitizes resistant MM to venetoclax

**Fig. 3:**
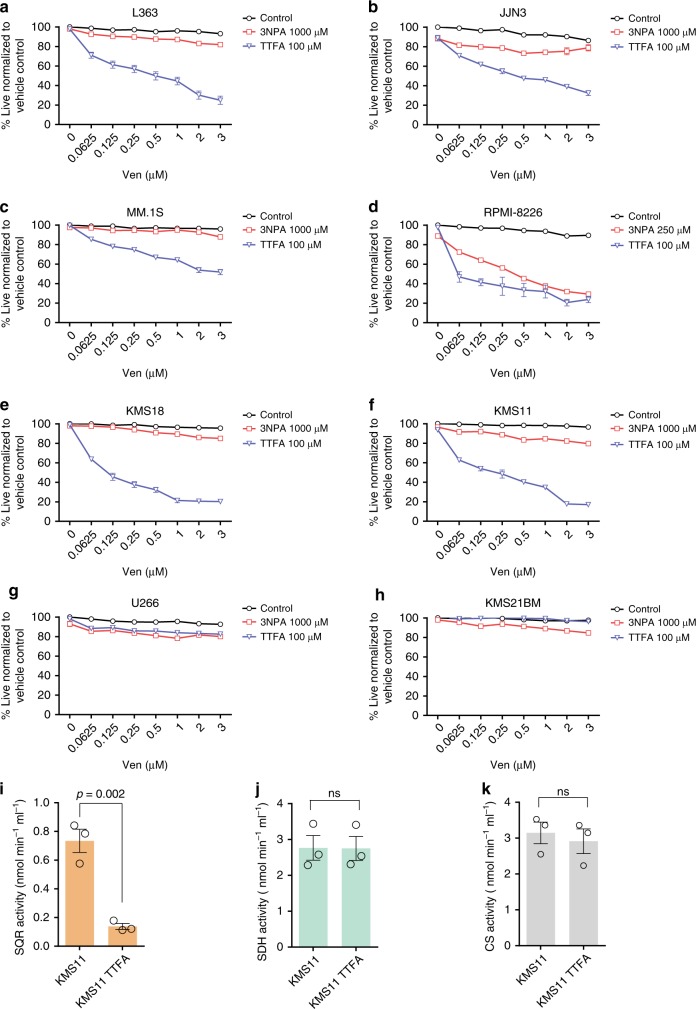
Inhibition of SQR with Qp site inhibitor TTFA effectively sensitizes resistant MM to venetoclax. **a**−**h** SQR inhibition with TTFA (100 μM) sensitizes indicated myeloma cell lines to venetoclax more effectively than inhibition of SDHA with 3-NPA (1000 μM except for RPMI-8226 treated with 250 μM) upon cotreatment with indicated doses of venetoclax for 24 h. Cell viability assessed by AnnexinV/DAPI staining. *n* = 3 independent experiments. Data are presented as mean values ± SEM. **i** SQR, **j** SDH and **k** CS activities were evaluated demonstrating selective inhibition of SQR activity upon 24 h TTFA treatment. Data are presented as mean values ± SEM. *p* values are calculated using a two-tailed unpaired Student’s *t* test. Data presented are from an *n* = 3 ± SEM. Source data are provided as a source data file.

Given the under studied and unique role of Complex II in directly connecting the TCA cycle to the ETC, we tested the impact of selectively inhibiting either the SDH or SQR activities of Complex II on venetoclax sensitivity. We tested the venetoclax sensitization effects of TTFA (SQR inhibitor) or 3NPA (SDH inhibitor) on eight venetoclax-resistant MM lines (Fig. 3a−h). 3NPA is a structural analog of succinate, which binds irreversibly with SDH inhibiting FAD reduction and fumarate production while TTFA is a direct Qp site inhibitor^28^. Inhibition of SQR in resistant MM lines, with the Qp site inhibitor TTFA, increased sensitivity to venetoclax in six of the eight lines tested (Fig. 3a−h). 3NPA on the other hand was less effective compared to TTFA in eliciting sensitivity to venetoclax. We also tested the efficacy of the TTFA−venetoclax cotreatment strategy in a colony-forming unit assay. MM lines (L363 and KMS11) were cultured in an agar matrix with single agents or the combination. Similar to results obtained in suspension culture, TTFA treatment sensitized MM lines to a lower dose of venetoclax (0.1 μM) reducing cellular viability (Supplementary Fig. 8). These results highlight the sufficiency of targeting the Qp site/quinone reductase activity of Complex II to induce BCL-2 dependence and sensitization to venetoclax. To demonstrate the selectivity of TTFA inhibiting SQR activity, we evaluated SQR, SDH, and CS activities in TTFA-treated KMS11 cells. We found significant reduction in SQR activity upon TTFA treatment (Fig. 3i), while SDH (Fig. 3j) and CS (Fig. 3k) activities remained unaffected. SRC was also concordantly reduced in the TTFA-treated KMS11 cells but not in unsensitized U266 and KMS21BM cells (Supplementary Fig. 9).

### SQR inhibition via Qp site SDHC-mutant introduction is sufficient to sensitize MM to venetoclax

**Fig. 4:**
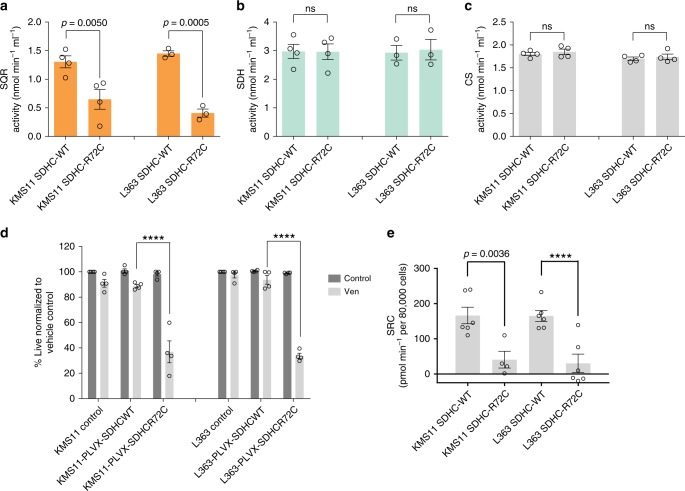
SQR inhibition via Qp site SDHC-mutant introduction sensitizes MM to venetoclax. **a** SQR, **b** SDH and **c** CS activities were determined in KMS11 and L363, SDHCKO cells expressing SDHC-WT or SDHC-R72C mutant constructs. *n* = 4 independent experiments except L363 SQR and SDH activity (*n* = 3). Data are presented as mean values ± SEM. Adjusted *p* values are calculated using a two-way ANOVA with post-hoc Sidak’s multiple comparisons test. **d** SDHC-WT and SDHC-R72C mutant-expressing cells ± 0.5 μM venetoclax (24 h) were evaluated for viability by AnnexinV/DAPI flow cytometric staining, demonstrating increased sensitivity of SDHC-R72C cells to venetoclax. *n* = 4 independent experiments. Adjusted *p* values are calculated using a two-way ANOVA with post-hoc Tukey’s multiple comparisons test. **e** Spare respiratory capacity determined in the indicated SDHC-WT and SDHC-R72C-mutant-expressing cells demonstrated a net reduction in SRC upon introduction of the mutant. *n* = 6 replicate Seahorse wells except KMS11 SDHC-R72C (*n* = 4). Data are presented as mean values ± SEM. Adjusted *p* values are calculated using a two-way ANOVA with post-hoc Sidak’s multiple comparisons test. **** denotes *p* value < 0.0001. Source data are provided as a source data file.

To further interrogate the sufficiency of SQR inhibition in inducing venetoclax sensitivity, we generated a Qp site variant of SDHC by mutating arginine to cysteine at position 72 which shows less binding affinity with ubiquinone^31^. We generated an SDHC guide RNA targeting an intronic region prior to the first SDHC exon so that we would not target the exogenously introduced SDHC-R72C mutant enzyme. We expressed the mutant in SDHC knockout (KO) L363 and KMS11 cell lines. This strategy was adapted as the SDHC KO cells did not survive in culture. As anticipated, cells expressing the SDHC-R72C mutant exhibited selective reduction of SQR activity (Fig. 4a) while maintaining SDH (Fig. 4b) and CS (Fig. 4c) activity  as compared to SDHC-WT cells. We also confirmed that Complex I activity was maintained in SDHC-R72C mutant-expressing KMS11 cells (Supplementary Fig. 10). The SDHC-R72C mutant sensitized KMS11- and L363-resistant MM cells to venetoclax (Fig. 4d), demonstrating that the Qp site and SQR inhibition is sufficient for inducing venetoclax sensitivity, despite maintenance of SDH, CS and Complex I activities. Since venetoclax-sensitive cells exhibited reduced mitochondrial respiratory parameters, we evaluated basal, maximal and coupled respiration in parental and SDHC-R72C mutant cells. Both KMS11 and L363 SDHC-R72C mutant cells exhibited a reduction in SRC (Fig. 4e), concordant with the loss of SQR activity. In summary, these results demonstrate that selective Complex II SQR inhibition increases BCL-2 dependence and venetoclax sensitivity.

### ATF4, BIM and NOXA regulate TTFA-induced venetoclax sensitivity in MM

**Fig. 5:**
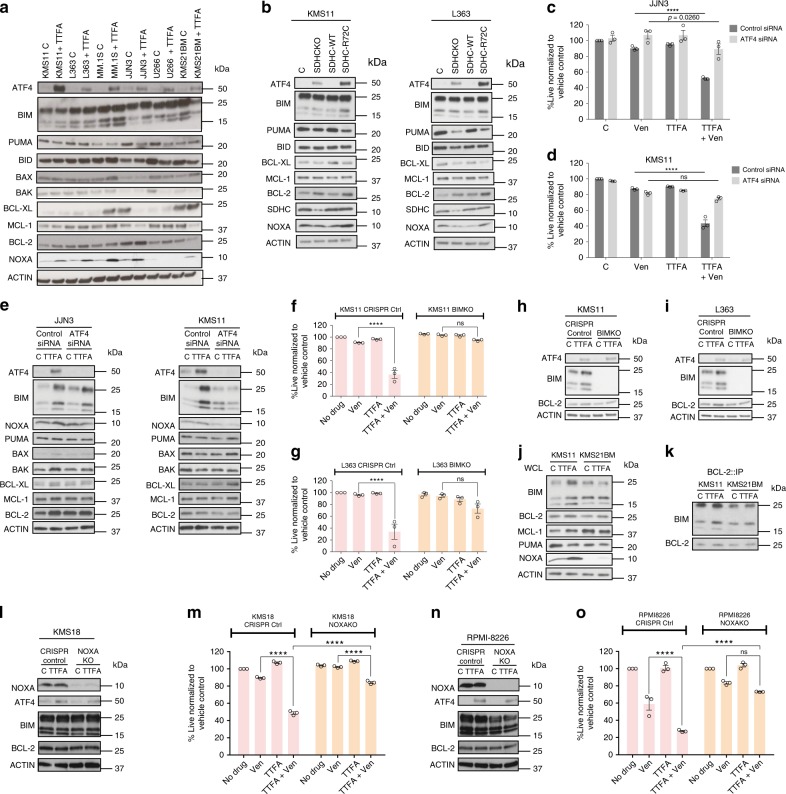
ATF4, BIM and NOXA regulate TTFA-induced venetoclax sensitivity in MM. **a** Expression of ATF4 and the indicated pro- and antiapoptotic proteins was evaluated in whole cell lysates of the indicated lines treated with ±100 μM TTFA for 24 h with Actin as loading control. Representative blots from one of three independent experiments is presented. **b** Protein expression levels were evaluated in SDHC-WT or SDHC-R72C mutant-expressing cells. **c**, **d** Control siRNA or ATF4 siRNA-transfected KMS11 or JJN3 cells were treated with venetoclax (0.5 μM), TTFA (100 μM) or the combination for 24 h and cell viability assessed by AnnexinV/DAPI flow cytometric staining. *n* = 3 independent experiments. Data are presented as mean values ± SEM. **e** Cells from (**c**, **d**) were used to prepare lysates for immunoblot evaluation of indicated proteins. **f**, **g** CRISPR Cas9 generated L363 and KMS11 BIMKO (KO efficiency shown in (**h**) and (**i**)) were treated with ±TTFA (100 μM), ±venetoclax (0.5 μM) for 24 h and cell death evaluated by Annexin V/DAPI flow cytometric staining. *n* = 3 independent experiments. Data are presented as mean values ± SEM. **j** Whole-cell lysates from KMS11 and KMS21BM treated or untreated with TTFA were evaluated for expression of indicated proteins by immunoblot analysis. **k** Cellular lysates from panel (**j**) were evaluated for immunoprecipitates of BCL-2 and bound BIM by immunoblotting. Representative blots from one of the three independent experiments is presented. **l**, **n** Whole-cell lysates from RPMI-8226 and KMS18 NOXA KO cells treated or untreated with TTFA were evaluated for expression of indicated proteins by immunoblot analysis. Representative blots from one of three independent experiments is presented. **m**, **o** RPMI-8226 and KMS18 NOXA KO cells were treated with ±TTFA (100 μM), ±venetoclax (0.5 μM) for 24 h and cell death evaluated by Annexin V/DAPI flow cytometric staining. *n* = 3 independent experiments. Data are presented as mean values ± SEM. Adjusted *p* values are calculated using a two-way ANOVA with post-hoc Tukey’s multiple comparisons test. **** denotes *p* value < 0.0001. Source data are provided as a source data file.

To identify the mechanistic basis for SQR inhibition-related venetoclax sensitivity, we evaluated the expression of ATF4, a transcription factor typically upregulated in response to cellular stresses such as ER stress, hypoxia and amino acid deprivation^32,33^. We also evaluated expression levels and binding properties of BCL-2 family members in SDHC-R72C mutants and resistant MM cells treated with and without TTFA. We detected increased expression of ATF4, NOXA, and a variable increase in BIM and/or BCL-2 protein expression in TTFA-treated MM cells (Fig. 5a). In addition, SDHC-R72C expressing mutants (Fig. 5b) exhibited a similar pattern of ATF4, BIM and BCL-2 induction. To interrogate the significance of ATF4 induction in venetoclax sensitization, we suppressed ATF4 expression in KMS11 and JJN3 cells using an ATF4 siRNA pool (KD efficiency demonstrated in (Fig. 5e) and the individual siRNAs (shown in Supplementary Fig. 11). Control and ATF4 siRNA-transfected cells were treated with or without TTFA and assessed for sensitivity to venetoclax. ATF4 KD significantly reversed venetoclax sensitivity, rescuing cell viability in venetoclax and TTFA cotreated cells (Fig. 5c, d and Supplementary Fig. 11) suggesting its role in elevating BCL-2 dependence. Importantly, TTFA-induced NOXA expression was reversed with ATF4 KD (Fig. 5e), while the induction of BIM and BCL-2 was variable. BIM induction was reduced in TTFA-treated KMS11 cells with ATF4 KD but maintained in TTFA-treated JJN3 cells (Fig. 5e). Knockdown of ATF4 also reduced the induction of BCL-2 and BAK in TTFA-treated JJN3 cells, identifying cell-context-specific contributory roles of other BCL-2 family proteins in elevating BCL-2 dependence in SQR-inhibited cells. The reduction in NOXA induction detected in the ATF4 KD cells (KMS11 and JJN3) treated with TTFA (Fig. 5e) suggests a role for NOXA in TTFA-induced venetoclax sensitivity. NOXA can displace BIM bound to MCL-1 to promote BIM binding to BCL-2 thereby increasing BCL-2 dependence in MM^7^.

To evaluate the role of BIM in TTFA-induced BCL-2 dependency, we generated a CRISPR/Cas9 knockout of BIM in KMS11 and L363 cells. BIM KO reversed the cytotoxic effect of cotreatment with TTFA and venetoclax in both KMS11 and L363 cells (Fig. 5f, g). This effect was observed despite the maintenance of ATF4 expression (Fig. 5h, i), suggesting it to be a downstream effector. These results confirm BIM as the major proapoptotic protein in TTFA/SQR inhibition-induced venetoclax sensitivity. We similarly tested the requirement for NOXA induction in TTFA-induced venetoclax sensitivity. Using NOXA KO lines (RPMI-8226 and KMS18), we demonstrate a lack of induction of NOXA in TTFA-treated NOXA KO as anticipated (Fig. 5l, n), and, importantly, a loss of TTFA-induced sensitivity to venetoclax (Fig. 5m, o). NOXA KO efficiency and expression levels of ATF4, BIM and BCL-2 were evaluated and are shown in (Fig. 5l, n).

Next, we investigated the regulation of ATF4, BIM and NOXA in MM cells that were not sensitized by TTFA treatment. We evaluated whole-cell lysates for total protein expression and performed coimmunoprecipitations to examine binding of BIM with antiapoptotic BCL-2 in KMS11 and the TTFA insensitive-t(11;14) KMS21BM cells. We excluded U266 from this study as it does not express NOXA^34^. We observed induction of BIM and NOXA upon TTFA treatment (Fig. 5j) only in KMS11 not in the TTFA insensitive-t(11;14) KMS21BM cells. Coimmunoprecipation of BCL-2 from control or TTFA-treated cells also demonstrated elevated BIM binding to BCL-2 in TTFA-treated KMS11 cells in contrast to the resistant KMS21BM cells (Fig. 5k). Cumulatively, these results identify NOXA and BIM to facilitate TTFA-ATF4-induced venetoclax MM sensitization.

### SQR activity inversely correlates with venetoclax sensitivity in MM patient samples

In order to clinically validate our observations, we utilized MM patient samples to (1) evaluate the impact of TTFA treatment on inducing sensitivity to venetoclax and (2) evaluate SQR activity in purified MM cells and its correlation with venetoclax cytotoxicity ex vivo and the response of patients being administered venetoclax (Trial NCT01794520: a Phase I Study Evaluating the Safety and Pharmacokinetics of venetoclax in Subjects with Relapsed or Refractory Multiple Myeloma). Complex I comprising of over 40 subunits is difficult to assay^35^. A direct evaluation of the NADH-ubiquinone oxidoreductase activity of Complex I requires release of Complex I from whole cells or manipulation of purified mitochondria as mitochondria are impermeable to NADH. On the other hand, SQR activity can be assessed in a fast and direct activity measurement assay using permeabilized cells. Due to the caveats associated with Complex I activity measurement and the simplicity of the SQR activity measurement assay, we further assessed SQR activity in purified CD138+ MM cells from patient samples.

**Fig. 6:**
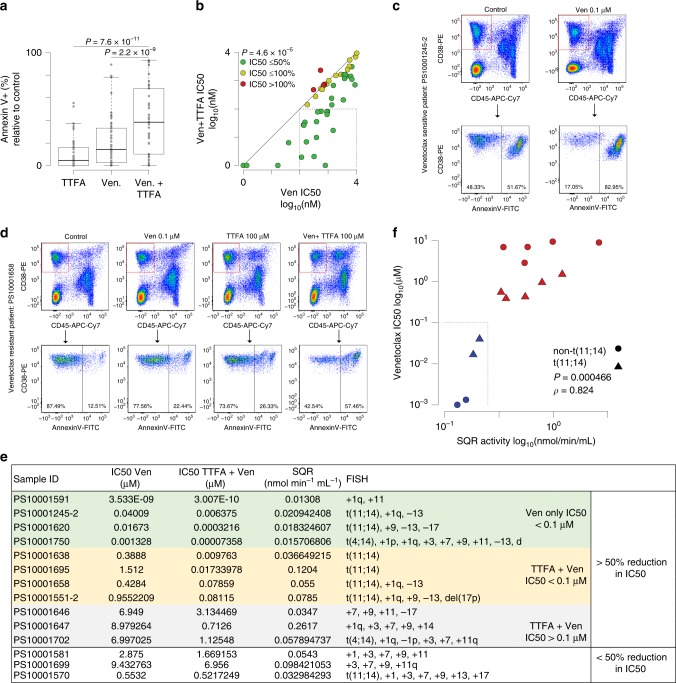
SQR activity inversely correlates with venetoclax sensitivity in MM patient samples. **a** Box plot of cell death measured by Annexin V staining relative to vehicle control in samples from 50 myeloma patient bone marrow aspirates treated with 0.1 μM venetoclax, 100 μM TTFA alone or in the combination for 24 h. CD38-PE and CD45-APC-Cy7 were used to gate myeloma cells. *n* = 50 biological independent samples. Boxplots show the median and quartiles with the whiskers extending to the most extreme data point within 1.5 times the interquartile range. IC50 ± TTFA was calculated as indicated in supplementary Table 1. **b** Scatter plot of IC50 for patient samples treated with venetoclax (Ven; *x*-axis) vs. those treated with Ven and 100 µM TTFA (*y*-axis). A diagonal line denotes one-to-one correspondence of IC50. Samples are colored by the change in IC50 relative to the Ven group (green: ≤50%; yellow: 50−100%; red >100%). The dashed box denotes patient samples with Ven IC50s > 100 nM and Ven + TTFA IC50s ≤ 100 nM. Samples with an IC50 < 1 nM are plotted at 1 nM. *p* values were calculated using a paired Student’s *t* test. **c**, **d** Flow plots of representative patient samples (exhibiting high and low SQR activity) and corresponding sensitivity of MM-gated cells to venetoclax and/or TTFA cotreatment are shown. **e** Ven ± TTFA IC50, SQR activity and FISH characteristics of purified CD138+ myeloma cells from 14 patient samples. Samples additionally segregated on the basis of >50% reduction in IC50. **f** Scatter plot of SQR activity and Venetoclax IC50 showing a positive correlation (Spearman’s rank correlation (*ρ*) = 0.824, *n* = 14, *p* value = 0.000466). Samples are colored by Ven IC50 (blue: ≤0.1 µM; red: >0.1 µM). Triangles denote t(11;14) samples and circles denote non-t(11;14) samples. The dashed box denotes patient samples with Ven IC50 ≤ 0.1 µM and SQR activity ≤ 0.25 nmol min^−1^ mL^−1^. Source data are provided as a source data file.

Bone marrow aspirate samples from 50 MM patients (characteristics reported in Supplementary Table 1) were treated with three doses of venetoclax (0.01, 0.1 and 0.5 μM) or TTFA (100 μM), or the combination for 24 h and cell death was assessed by AnnexinV staining. These results show that the combination of TTFA plus 0.1 μM venetoclax resulted in significantly more cell death in 50 primary myeloma cells (Fig. 6a). A venetoclax IC50 <0.1 μM is characterized as a sensitive sample based on previous studies in MM that evaluated venetoclax IC50 in relation to sensitivity^36,37^. By this criteria 15 of 50 (30%) samples were sensitized by TTFA treatment (Supplementary Table 1). However, comparison of the IC50 of venetoclax plus TTFA vs. venetoclax alone showed that 46 of 50 (92%) had increased venetoclax sensitivity with 31 (62%) showing more than a 50% decrease in IC50 (Fig. 6b). Of the 31 samples where TTFA did reduce their IC50 by at least 50%, 11 exhibited the t(11;14) translocation (Supplementary Table 1). TTFA sensitized MM cells to venetoclax, increasing cell death in CD38+ CD45− gated MM cells (Fig. 6c, d) with no to minimal impact on viability of the non-MM cells contained within the aspirate (Supplementary Fig. 12), suggestive of the selective synthetic lethality of this combination in MM cells.

We were also able to assess SQR activity in purified CD138+ MM cells from 14 samples (SQR activity, venetoclax IC50 and patient sample characteristics presented in Fig. 6e). There was a positive correlation between SQR activity and venetoclax insensitivity/resistance ex vivo (Spearman’s rank correlation, *ρ* = 0.824, *p* < 0.001), validating our observations in cell lines where low SQR activity correlates with venetoclax sensitivity (Fig. 6f). We found low SQR activity (i.e. <0.25 nmol min^−1^ mL^−1^) in four patient samples, including two non-t(11;14) (PS10001591 and PS10001750), to correspond with ex vivo venetoclax sensitivity i.e. venetoclax IC50 < 0.1 μM. In addition, three patients PS10001245-2 and PS10001620 which were t(11;14) and PS10001591, a non-t(11;14), either on the M15-538 trial (NCT02899052) or off trial were found to have low SQR activity and a clinical response (as described in Supplementary Table 1).

On the other hand, among the ten resistant samples (based on ex vivo sensitivity), all having high SQR activity, five exhibited the t(11;14) translocation, highlighting the ability of SQR activity to identify venetoclax-resistant MM among t(11;14) MM. One of these patients (PS10001551-2) was a post-venetoclax treatment refractory sample, the other (PS10001658) failed to achieve an objective response and the third (PS10001570) was responsive, as reported in Supplementary Table 1. While all ten resistant samples showed increased sensitivity to venetoclax upon TTFA treatment, seven samples showed greater than 50% reduction in IC50 values. Importantly, PS10001551-2 that showed more than tenfold reduction in IC50 was the t(11;14) patient who relapsed on venetoclax and was additionally refractory to other therapies. This patient’s MM cells exhibited high SQR activity highlighting (1) SQR activity correlates with a refractory patient response (Supplementary Table 1) and the ability of SQR inhibition to induce venetoclax sensitization. While our sample size for correlation with patient response is small, we believe the correlation of SQR activity with patient response warrants further investigation.

### Inhibition of Complex I and distal ETC Complexes sensitize MM to venetoclax

**Fig. 7:**
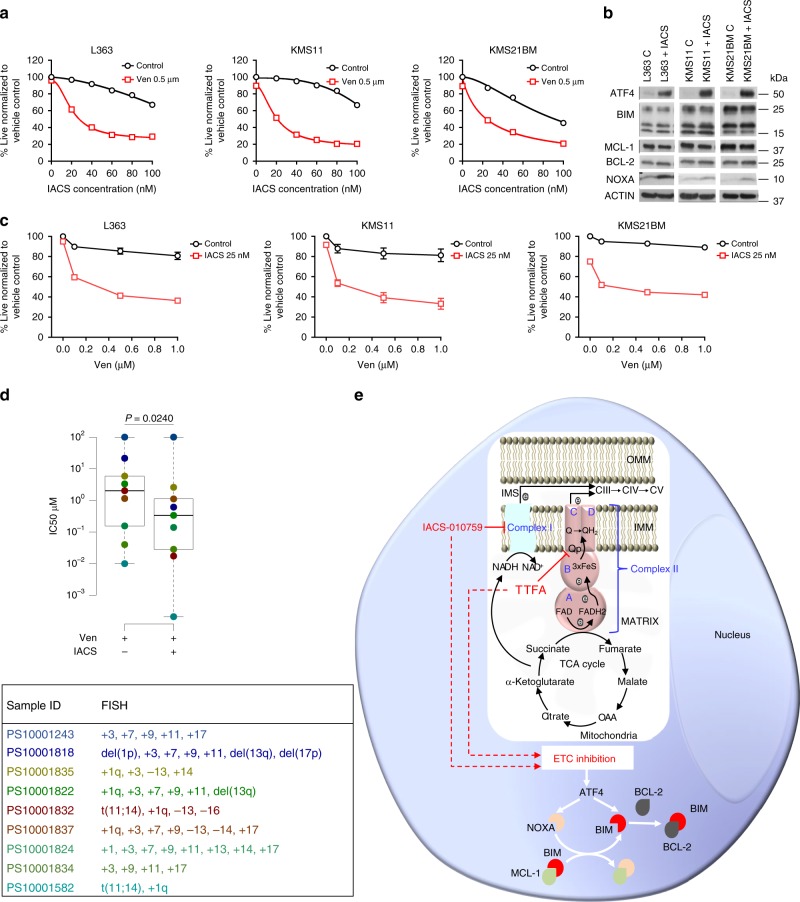
Inhibition of Complex I with IACS-010759 sensitizes resistant MM to venetoclax. **a** Dose−response curves for cotreatment of indicated cell lines with 0.5 µM venetoclax and increasing doses of IACS-010759 for 24 h. Cell viability assessed by AnnexinV/DAPI flow cytometric staining. **b** Expression of ATF4 and the indicated pro- and antiapoptotic proteins were evaluated in whole-cell lysates of the indicated lines treated ±25 nM IACS-010759 for 24 h. Actin was assessed as a loading control. Representative blots from one of two independent experiments is presented. **c** Dose−response curves for cotreatment of 25 nM IACS-010759 with increasing doses of venetoclax. Cell viability assessed by AnnexinV/DAPI staining. *n* = 3 independent experiments. Data are presented as mean values ± SEM. **d** Box plot of IC50 values of Ven and Ven + IACS, and table with FISH characteristics of nine myeloma patient samples. *n* = 9 biologically independent samples. Boxplots show the median and quartiles with the whiskers extending to the most extreme data point within 1.5 times the interquartile range. PS10001243 was resistant to Ven ± IACS and has been represented to have an artificial IC50 of 100 µM for Ven ± IACS in the box plot. **e** Mechanistic representation of how Complex I and Complex II regulate BCL-2 dependence in an MM cell. IACS-010759 and TTFA inhibit Complex I and Complex II, respectively, resulting in ETC inhibition. ETC inhibition regulates BCL-2 dependency by inducing ATF4. ATF4 induces NOXA (light orange) that displaces BIM (red) from MCL-1 (green). The increased binding of BIM to BCL-2 (gray) elevates BCL-2 dependence leading to sensitization to venetoclax. Source data are provided as a source data file.

To further inquire whether Complex I inhibition could also sensitize MM cells to venetoclax, we tested a Complex I inhibitor (IACS-010759^38^, currently in phase I clinical trials for relapse refractory AML (NCT02882321) and relapse refractory lymphoma and solid tumors (NCT03291938)). Treatment of three representative resistant lines (KMS11, L363 both non-t(11;14); and KMS21BM a t(11;14) line) with increasing doses of IACS-010759 sensitized cells to 0.5 μM venetoclax after a 24 h treatment (Fig. 7a). Further, Complex I inhibitor piericidin also sensitized resistant MM cell lines to venetoclax (Supplementary Fig. 13a, b). Western blot analysis of cells treated with 25 nM IACS-010759 for 24 h revealed induction of ATF4 and NOXA suggesting that, similar to TTFA, IACS-010759 also sensitizes MM cells to venetoclax through regulation of ATF4 and NOXA (Fig. 7b). The dose of IACS-010759 was selected based on proximity to the IC50 of IACS-010759 in combination with 0.5 μM venetoclax and minimal impact of single-agent IACS-010759 on viability after a 24 h treatment (Fig. 7a). Cotreatment with 25 nM IACS-010759 significantly reduced the IC50 of venetoclax in the tested cell lines (Fig. 7c). We next tested if inhibition of Complex III, IV and V could also sensitize resistant MM to venetoclax. As seen with Complex I and SQR inhibition, inhibition of Complex III, IV and V by Complex III inhibitor (antimycin), Complex IV inhibitor (sodium azide) and Complex V inhibitor (oligomycin) at doses which reduced ATP levels by about 50% after 24 h (Supplementary Fig. 13c, d) sensitized L363, KMS11 and KMS21BM to venetoclax (Supplementary Fig. 13e−g).

Lastly, we tested the efficacy of IACS-010759 in sensitizing MM patient samples to venetoclax. Details of the patient samples and IC50 values are summarized in Supplementary Table 2. We found that eight out of nine samples showed increased sensitivity with IACS and there was >50% reduction in the IC50 of venetoclax in five of the samples (Supplementary Table 2). Interestingly, out of the two t(11;14) patient samples (PS10001582 and PS10001832), PS10001832 was resistant to venetoclax alone and was significantly sensitized on treatment with IACS-010759 (IC50 of Ven + IACS = 0.01732 µM). This suggests that IACS-010759 can be used to sensitize non-t(11;14) MM and venetoclax-resistant t(11;14) patients, only 40% of which are responsive to single-agent venetoclax. These results are summarized in Fig. 7d. Additionally, similar to TTFA, combined treatment of IACS-010759 with venetoclax had minimal impact on the viability of non-MM cells contained within the aspirate (Supplementary Fig. 14). In sum, IACS-010759 reduced the IC50 for venetoclax and sensitized MM patient samples to single-agent venetoclax providing translational impetus for testing this combination in MM.

To summarize our findings, a model for the mechanistic basis for induction of BCL-2 dependence with IACS-010759 and TTFA is presented in Fig. 7e.

## Discussion

MM cells expressing the t(11;14) translocation afford a unique opportunity to investigate the intrinsic mechanisms responsible for BCL-2 dependence and identify biology that can be harnessed to increase venetoclax sensitivity in the broader resistant MM population. We were specifically interested in determining whether metabolism and cellular energetics played a role in the increased BCL-2 dependence and sensitivity of t(11;14) MM to venetoclax.

The primary translocations in myeloma occur early in the evolution of the disease and the t(11;14) translocation juxtaposes the *cyclin D1* gene to the immunoglobulin heavy chain (*IgH*) gene enhancer resulting in dysregulated cyclin D1 expression^39^. Cyclin D1-dependent activation of CDK4/6 promotes G1-S phase cell cycle transition. Cyclin D1 also exhibits a number of CDK-independent functions by regulating more than 35 transcription factors to block cellular differentiation, proliferation, mitochondrial biogenesis and mitochondrial oxidative phosphorylation^40^. While our CoMMpass data analysis does not have matching venetoclax sensitivity data making it impossible to identify a venetoclax-sensitive gene signature, the observation of reduced ETC gene expression in t(11;14) vs. non-t(11;14) suggests that ETC activity may be a biomarker of venetoclax sensitivity. We have used t(11;14) as a proxy as ~40% of t(11;14) patients are venetoclax-sensitive whereas <6% of non-t(11;14) are venetoclax-sensitive^37,41^. This is supported by our functional studies in cell lines that demonstrated reduced ETC activity to be critical to venetoclax sensitivity. Our study has illuminated that low SQR activity is a reliable read-out of venetoclax sensitivity in all MM cells, irrespective of t(11;14) status. Importantly, SQR activity can be evaluated in intact permeabilized whole cells in a fast and direct activity measurement assay. Direct assessment of the ubiquinone reductase activity of Complex I is technically challenging as it requires isolation of mitochondria followed by permeabilization while maintaining the structure and activity of Complex I^35^. SQR activity on the other hand can be determined in semi-permeabilized whole cells supplemented with exogenous succinate.

We also sought to understand whether an elevation in OXPHOS promotes resistance to venetoclax. We have previously demonstrated that glutamine deprivation (that suppresses OXPHOS) sensitizes resistant MM cell lines and patient samples to venetoclax^23^. Importantly, this sensitization to venetoclax was reversed upon supplementation of the glutamine-deprived cells with cell permeant α-ketoglutarate, suggesting that restoration of OXPHOS promotes resistance to venetoclax^23^. Interestingly, recent studies in venetoclax-relapsed CLL also demonstrate changes in cellular metabolism and an elevation in OXPHOS to promote venetoclax resistance^42^, supporting our observations of elevated OXPHOS correlating with reduced BCL-2 dependence.

The dependence of B cells on specific prosurvival BCL-2 members is activation, development and differentiation stage specific. The activation/differentiation of normal murine B cells is associated with increased ABT-737 (a Bcl-2, Bcl-xL, and Bcl-w antagonist) sensitivity due to a decline in BCL-2 and increase in BCL-xL expression upon differentiation to a plasma cell^43^. Additionally, prior studies testing the effects of ABT-737 on the murine immune system showed ABT-737 to inhibit the establishment of newly arising bone marrow plasma cells and memory B cells with no effect on pre-existing plasma cells, or germinal center B cells^44^. These results suggest that in B cells, BCL-2 dependence is regulated in an activation and/or differentiation-stage-specific manner. With respect to metabolism, B-cell activation and differentiation to a plasma cell are associated with an increased dependency on the TCA and OXPHOS^45,46^. t(11;14) MM are differentiated antibody-producing plasma cells. However, early studies examining expression profiles of CD138+ MM in the CD2 subgroup of CCND1/CCND2-amplified MM cells identified elevated expression of B-cell lineage markers like MS4A1/CD20, VPREB and PAX5^47^. Thus, a multiple myeloma plasma cell malignancy which should be exhibiting elevated OXPHOS may get locked into metabolically compromised suppressed ETC state upon expressing/maintaining B-cell-like features. This suppression of the ETC activity is thus also associated with the sensitivity to venetoclax (Fig. 1a), allowing for a potent strategy of synthetic lethality with venetoclax. Interestingly, the non-t(11;14) OCI-MY5 line that exhibits low SQR activity and venetoclax sensitivity is also likely to be more B-cell-like or undifferentiated based on its high expression of CD20 (VA Gupta and LH Boise, unpublished observations). SQR activity in the limited number of DLBCL lines we assessed was low and we were unable to detect a correlation between low SQR activity and venetoclax sensitivity, again supportive of a differentiation-specific component to ETC activity and BCL-2 dependence.

Our studies have allowed us to also unravel the ETC as a target for venetoclax sensitization. Our initial studies focused on Complex II. SDH is unique in that it is the only ETC complex with all four subunits being solely nuclear encoded and exclusively positioned to function in both the TCA and ETC. The four SDH subunits have distinct roles with mutations in SDHA (albeit rare) linked to impaired oxidative phosphorylation and severe metabolic disorders^48^ while mutations in SDH B, C and D are linked to tumorigenesis when present with Complex I deficiency^49^. Disruption of SQR activity with the introduction of the SDHC R72C mutant^31^ or with TTFA treatment that disrupts the SQR activity of the enzyme without impacting SDH activity or Complex I activity sensitizes MM cells to venetoclax, underscoring the sufficiency of targeting SQR to induce BCL-2 dependence. Previous work has shown that death-resistant cells have increased SRC^50^ and Complex II plays a role in maintaining SRC and resistance to death^51^. Here we show that venetoclax-resistant MM exhibit significantly higher SRC than venetoclax-sensitive MM and inhibition of SQR by TTFA or the R72 SDHC mutant lowers the SRC of venetoclax-resistant MM and sensitizes them to venetoclax. Our observations thus warrant the development of selective potent SQR inhibitors for creating a heightened dependence on BCL-2 and venetoclax sensitivity. Studies in AML have shown that targeting Complex II with azacitidine induced venetoclax sensitivity^52^. Previous studies have demonstrated that the oncometabolite (R)-2-HG expressed in IDH 1/2 mutant-expressing AML induces BCL-2 dependence by suppressing cytochrome c oxidase (Complex IV)^53^ While we subsequently show that targeting the other ETC complexes can also sensitize to venetoclax, the unique aspect of Complex II in not contributing to proton transport but being a conduit of electrons from succinate to labile ubiquinone, whose inhibition can generate signals that can elevate BCL-2 dependence, are worthy of further exploration.

Complex II is linked to the generation of ROS, accumulation of succinate and the induction of HIF1-α^54^ that can lead to induction of ATF4^55^. ATF4 is post-translationally induced as part of the integrated stress response to anoxia, hypoxia, proteasome inhibition or nutrient deprivation, upregulating genes that sustain amino acid metabolism and anti-oxidant responses^56^. ATF4 has both prosurvival effects through the activation of autophagy^33^ and proapoptotic effects through the induction of C/EBP homologous protein (CHOP) which in turn induces BIM^57^, NOXA^58^ and PUMA^59^. NOXA has previously been implicated in inducing BCL-2 dependence and sensitivity to venetoclax^36^, implicated in bortezomib cytotoxicity in MM^60^ and in panobinostat sensitivity of DLBCL^61^. ATF4 in specific cellular contexts can induce NOXA^62,63^ which, in binding MCL-1, can increase the quantity of BIM bound to BCL-2, potentially explaining TTFA-induced BCL-2 dependency. We must note that U266 cells do not express basal NOXA^34^ and NOXA was not induced with TTFA treatment in U266 which could contribute to its reduced sensitivity to BCL-2 antagonists. However, interestingly, while both ATF4 and NOXA are induced in KMS21BM upon treatment with TTFA and IACS-010759, 100 μM TTFA does not sensitize these cells to venetoclax. This can be explained by the difference in the levels of induction of ATF4 and NOXA proteins upon Complex I and Complex II inhibition. Concordantly, the higher level of ATF4 and NOXA induction upon IACS-010759 treatment is enough to sensitize KMS21BM to venetoclax. On the other hand, the low level of NOXA induction in case of TTFA treatment of KMS21BM is not sufficient to increase BIM binding on BCL-2 (Fig. 5k) and sensitize the cells to venetoclax treatment.

Studies from the Letai group have clearly established that refractory cancers, including MM and other cancers subject to prior chemotherapy, exhibit reduced “priming” i.e. exhibit suboptimal quantities of BH3 activators bound to antiapoptotics, increasing the threshold required to induce apoptosis^64–67^. Hence, approaches that can increase the “primed” state of the cell will not only increase sensitivity to existing chemotherapy but also enhance sensitivity to BH3 mimetics. Normal cells, on the other hand, being further removed from the apoptotic threshold are not sensitized by ETC inhibition and venetoclax cotreatment, further supported by our observations on the insensitivity of non-MM cells to the venetoclax IACS-010759/TTFA + venetoclax treatment. Several strategies (dexamethasone^36,37^ with or without a proteasome inhibitor (NCT02755597) for MM, hypomethylating agents in AML^13,52,68^) are being tested to increase BCL-2 dependence and sensitivity to venetoclax. It is the presence of minimal residual disease (MRD) that is well connected to the emergence of refractory disease. There continues to be an unmet need in achieving deeper responses in t(11;14) patients to remove MRD and reduce toxicity. Therefore, we believe that identification of novel targets to achieve sensitization even in t(11;14) patient samples is important. The isolated myeloma patient samples, in contrast to cell lines, are not proliferating ex vivo. It is possible that proliferating cells (more characteristic of poor prognosis MM^47^) are more dependent upon OXPHOS due to energetic and biosynthetic requirements of cell division and thus could make them more sensitive to targeting ETC activity. Given the genetic heterogeneity of MM, targeting the ETC has the potential to maximize the clinical application of the highly potent BCL-2 antagonist, venetoclax, and provide a more unifying approach to tackle refractory resistant MM.

## Methods

### Chemicals and reagents

Standard chemicals, 2-thenoyltrifluoroacetone (TTFA; #T27006), rotenone (#R8875), sodium azide BioXtra (#S8032), antimycin A (#A8674), dimethyl malonate (#04011), d-α tocopherol succinate (#T3126), 2,6-dichloroindophenol sodium salt hydrate (DCPIP; #D1878), phenazine methosulphate (PMS; #P9625), MTT (#M5655), acetyl CoA (#A2181), 5,5′-dithiobis(2-nitrobenzoic acid) (DTNB; #D218200), bovine serum albumin (#A6003), doxycycline hyclate (#D9891), 3-Nitropropionic acid (#N5636) and oxaloacetic acid (#O4126) were purchased from Sigma-Aldrich. Carbonyl cyanide p-trifluoro-methoxyphenyl hydrazone (FCCP; #BML-CM120-0010) was purchased from Enzo Life Sciences. Venetoclax (#2253-1) and IACS-010759 (#B2231-1) were purchased from Biovision Inc. Potassium phosphate monobasic (KH_2_PO_4_; #BP362-500) and ethylenediamine tetra acetic acid (EDTA; #BP118-500) were purchased from Fisher Scientific. Decylubiquinone was purchased from Abcam (#ab145233). Oligomycin was purchased from Merck Millipore (#495455). Triton X-100 (0694) was purchased from Amresco. Polybrene (#TR-1003-G) was purchased from Millipore. Puromycin (#631306) was purchased from Clontech.

### Cell lines

MM cell lines were obtained from the following sources: KMS18 (Dr. L Bergsagel, Mayo Clinic, AZ), RPMI-8226 (ATCC), U266, L363, KMS11, JJN3 (Dr. M. Kuehl, NCI, MD), MM.1S (Dr. S. Rosen, City of Hope, CA), KMS18, KMS21BM, KMS12BM, KMS12PE and KMS27 from JCRB Japanese Collection of Research Bioresources and KARPAS-620, OCI-MY5 and H1112 from Jonathan Keats, TGEN, City of Hope affiliate. DLBCL cell lines were obtained from the following sources: OCI-LY-19, OCI-LY-10 and HBL-1 from Dr. C. Henry; SUDHL-4, SUDHL-6 and U2932 from Dr. L. Mizrachi-Bernal, Emory University, Atlanta, GA. HEK 293T cells were obtained from American Type Culture Collection.

### Cell culture

MM cell lines were routinely cultured in complete RPMI-1640 (Corning #10-040-CV) with 10% Fetal Bovine Serum (FBS, DLBCL lines in 20% FBS), HEK293T cells in Dulbeccoʼs Modified Eagle Medium (DMEM, Sigma #D5030) with 10% FBS and 100 U per mL penicillin, 100 µg per mL streptomycin and maintained in a 37 °C incubator with 5% CO_2_.

### Immunoblotting

Cells were harvested and washed twice with phosphate-buffered saline (PBS) and whole cell lysates were prepared with RIPA buffer supplemented with phosphatase and protease inhibitors and 1% phenylmethylsulfonyl fluoride (PMSF) for 30 min on ice. Debris was removed by centrifugation at 14,000 rpm at 4 °C for 15 min. Total protein concentration was determined using Bio-Rad Protein Assay Dye Reagent Concentrate (#5000006). Samples were normalized to a total protein concentration of 20−30 µg and resolved using sodium dodecyl sulfate-polyacrylamide gel electrophoresis (SDS-PAGE) (Bio-Rad #4568096, #4568093) after being boiled at 95 °C for 5 min. Proteins were transferred to polyvinylidene fluoride (PVDF) membrane (Millipore # IPVH00010). Antibodies to: BCL-x_L_ (#2764), BIM (#2933), PUMA (#4976), BAX (#2772), BID (#2002), and BCL-2 (#4223) were obtained from Cell Signaling; MCL-1 (#sc-819), SDHA (F-2) (sc-390381), SDHB (G-10) (#sc-271548), SDHC (C-2) (#sc-515102) and NDUFS2 (B-3) (#sc-390596) from Santa Cruz Biotechnology; ATF4 (#ab23760) from Abcam; beta-actin (#A5441), BAK (#06-536) and NOXA (#OP180) from Sigma-Aldrich. Antibody to BCL-2 (Hamster) used in co-IP was purchased from BD-Pharmingen (#51-1513GR). PVDF membranes were incubated in blocking solution (5% milk in tris-buffered saline with tween TBST) for 1 h followed by washing and incubation with primary antibodies (1:1000 dilution made in BLOK™ Casein in TBS (G-Biosciences, #786-196)) overnight. Secondary antibodies were used at a dilution of 3:10,000 in blocking solution for 1 h.

### Coimmunoprecipitation

Cells were washed once with PBS and lysed in 3-((3-cholamidopropyl) dimethylammonio)-1-propanesulfonate (CHAPS) buffer containing protease and phosphatase inhibitors and PMSF for 30 min on ice. Debris was removed by centrifugation at 14,000 rpm at 4 °C for 15 min. Total protein concentration was determined using Bio-Rad Protein Assay Dye Reagent Concentrate (#5000006). 340 µL lysates containing 113 µg protein were precleared by incubating for 1 h at 4 °C with 50 µL of Protein G Agarose (Millipore#16-266), prewashed with PBS and CHAPS buffer. Precleared lysates were collected by centrifugation at 5000 rpm for 2 min at 4 °C and incubated with rotation overnight at 4 °C with 50 µL of Protein G Agarose, preincubated with 7 µL of BCL-2 (CST #4223S) antibody overnight. Pelleted matrix was washed twice with 500 μL ice-cold PBS, and proteins were eluted after boiling at 95 °C for 5 min in 30 µL of 2× electrophoresis sample buffer containing β-mercaptoethanol.

### Cell viability assays

For cell viability assays, 0.125 × 10^6^ cells per mL were treated with the indicated concentration of drug and evaluated for viability by AnnexinV/DAPI flow cytometric analysis. Cells were harvested and washed twice with PBS and stained with Annexin V–fluorescein isothiocyanate (BD-Pharminogen# 556419) and DAPI according to the manufacturer’s instructions. Samples were acquired on an FACSCanto II RUO Special Order System flow cytometer from BD (Becton Dickinson, San Jose, CA) and analyzed with the De Novo Software FCS Express Version 4.

### Isolation and purification of primary myeloma cells

Bone marrow aspirates or peripheral blood samples from consenting myeloma patients were diluted to 25 mL with PBS and over laid on lymphocyte separation media (Corning). Following centrifugation, the collected buffy coat was washed with PBS and resuspended in culture medium. Cells subject to various treatments were stained with anti-CD38-phyocerythrin and anti-CD45-allophycocyanin-Cy7 (BD Biosciences) to identify MM cells^69^. All samples were collected following an Emory University Institutional Review Board (IRB)-approved protocol in compliance with all relevant ethical regulations. The authors affirm that human research participants provided informed consent to participate in the study (IRB00057236). CD138+ cells were purified using MACS Miltenyi Biotec CD138+ human microbeads as per instructions (#130-051-301). IC50s of venetoclax alone or in combination with TTFA/IACS-010759 in myeloma samples were calculated using nonlinear regression (curve-fit) analysis under agonist vs. normalized response with variable slope using GraphPad Prism 5.

### Enzyme activity assays

Succinate ubiquinone reductase (SQR) and succinate dehydrogenase (SDH) activity assays were performed by absorbance-based assays using different electron acceptors. 0.3 × 10^6^ cells were harvested per sample, washed with PBS and resuspended in buffer containing 10 mM KH_2_PO_4_ (pH 7.4), 2 mM EDTA and 1 mg per mL BSA, 10 mM sodium azide, 5 μM rotenone, and 2 μM antimycin (assay buffer). Control, 50 µM TTFA and 5 mM malonate treated samples were supplemented with 20 mM succinate. Sample without succinate was used as a negative control for the assay. For SQR assay, reaction was initiated by adding reaction buffer containing 80 µM decylubiquinone and 80 µM DCPIP (molar extinction coefficient = 19.1 mM^−1^cm^−1^). Reduction in the absorbance of DCPIP was monitored at 600 nm every min for 10 min using a BioTek SYNERGY H1 microplate reader. For SDH assay, reaction was initiated by adding reaction buffer containing 400 µM phenazine methosulphate (PMS) as an exogenous electron carrier and 150 µM MTT (molar extinction coefficient = 13 mM^−1^ cm^−1^). Change in the absorbance of MTT was monitored at 570 nm for 10 min at 1 min intervals. Citrate synthase activity assay was measured in 0.3 × 10^6^ cells harvested per sample and washed with PBS. Cells were resuspended in 10 mM Tris (pH 8.5) and 0.1% Triton X-100 assay buffer, containing 300 µM acetyl CoA and 100 µM 5,5′-dithiobis(2-nitrobenzoic acid) (DTNB = molar extinction coefficient 13.6 mM^−1^ cm^−1^). Reaction was started by the addition of 0.5 mM oxaloacetic acid and change in absorbance recorded at 412 nm wavelength every min for 10 min^70^. SQR, SDH and CS activity measurements were performed with 100 µL sample containing 0.3 × 10^6^ permeabilized cells. Complex I activity assay was performed using Abcam (ab109721) Complex I Enzyme Activity Microplate Assay Kit (Colorimetric), per instructions. Complex I specific antibodies are precoated in the microplate wells where target was immobilized. Complex I activity was determined by following the oxidation of NADH to NAD+ and the simultaneous reduction of the provided dye (*ε* = 25.9 mM^−1^ well^−1^) which leads to increased absorbance at OD 450 nm. Complex I activity measurements were perfomed with 250 µg total protein per sample resuspended in 200 µL assay buffer. Enzyme activities were calculated from the change in absorbance using the extinction coefficients of the respective dyes.

### Oxygen consumption rate

Basal, maximal and coupled respiration were determined by measuring OCR using Seahorse bioscience extracellular flux (XFe96) analyzer^71,72^. Myeloma cells were cultured in RPMI-1640 medium treated or untreated with TTFA for 24 h in a six-well plate. Harvested cells were washed with PBS and plated at 80,000 cells per well in 5−8 replicates in Cell-Tak (Becton Dickinson)-precoated 96-well plates custom designed for XFe96 analysis following the manufacturer’s recommendations. OCR was evaluated over time after injection of oligomycin (final concentration 2.5 µM), carbonyl cyanide p-trifluoro-methoxyphenyl hydrazone (FCCP; final concentration 0.5 µM), and antimycin and rotenone (final concentration 2 µM each). The results from the mitochondrial respiration assay were analyzed by the XFe wave software (Seahorse Bioscience Inc., MA), and displayed as OCR (pmol min^−1^ per 80,000 cells).

### Generation of CRISPR KO cells and siRNA knockdown

Crispr/Cas9 BIM and SDHC knockout (KO) cell lines were generated by designing sgRNAs using the http://crispr.mit.edu/ and http://www.broadinstitute.org/rnai/public/analysis-tools/sgrna-design-v1 web tools. sgRNA for SDHC was designed targeting the intron region just before the Exon 1 of *sdhc*. “CACCG” was added towards the 5′ end of 20-nt guide sequence. “AAAC” was added at the 5′ end and an extra “C” towards the 3′ end of the opposite pair of sgRNA. sgRNA pairs were annealed and cloned in LentiCRISPRv2 vector at the *Bsm*bI site according to GecKO-Zhang’s lab Lentiviral CRISPR Tool box. The following primers were used for inserting SDHC guides into LentiCRISPRv2 vector: CACCGGTCCAGACCGGAACCCAAGA (forward) and AAACTCTTGGGTTCCGGTCTGGACC (reverse). The following primers were used for inserting BIM guides into LentiCRISPRv2 vector: CACCGGTAGACAATTGCAGCCTG (forward) and AAACCAGGCTGCAATTGTCTACC (reverse). Vector was digested with NEB *Bsm*BI restriction enzyme, dephosphorylated with CIP (alkaline phosphatase) and purified using QIAGEN Gel extraction kit as per the manufacturer’s protocol^73–75^. After ligation in LentiCRISPRv2, clones were transformed in *Escherichia coli* Stbl3 competent cells (Invitrogen# C737303). Plasmids were purified from ampicillin-resistant colonies and sequence confirmed by Sanger sequencing using U6F primer probe. LentiCRISPRv2-sgRNA plasmids were cotransformed with psPAX2 (packaging plasmid) and pMD2.G (VSV-G expressing plasmid, envelope plasmid) in HEK293T cells using jet-PRIME Polyplus transfection reagent to generate virus. Lentiviral particles were transduced in MM cell lines using a standard transduction protocol. Briefly, 1.5 × 10^6^ cells were plated per well in a six-well plate in UltraCulture serum-free medium (Lonza #12-725F) containing 2.5 µg per mL Polybrene Infection/Transfection Reagent (Sigma-Aldrich #TR-1003-G). Spinfection was performed by adding 500 µL of virus solution per well followed by centrifugation at 2500 rpm at room temperature for 1.5 h. The plate was then placed in a 37 °C CO_2_ incubator for 2 h. Cells were harvested and washed with PBS and resuspended in regular RPMI growth medium and allowed to grow for 72 h. Single-cell clones were generated from cells sorted in 96-well plates. ATF4 siRNA knockdowns were generated using Dharmacon ON-TARGETplus Human ATF4 siRNA pool (L-005125-00-0005) and individual siRNAs from the deconvoluted pool by transfection using V-reagent as per the manufacturer’s protocol.

To generate NOXA KO lines, inducible Cas9 expressing cells were generated using the pCW-Cas9 plasmid through lentiviral infection. Virus was produced by transfecting HEK293T cells with pCW-Cas9 (gift from Eric Lander and David Sabatini: Addgene plasmid # 50661) and packaging plasmids DR8 and VSVg using lipofectamine 2000. Viral supernatant was collected at 48 h, filtered, and supplemented with 4 µg per mL polybrene. Myeloma cells were resuspended in viral supernatant and centrifuged at 1000 × *g* and 37 °C for 90 min. Infected cells were selected with puromycin for 1−2 weeks before single-cell cloning by limiting dilution. Clones were screened for inducible Cas9 by adding doxycycline at 1 µg per mL for 24 h followed by lysis, SDS-PAGE, and western blot with anti-FLAG antibodies. NOXA CRISPR knockout was achieved by cloning a published sgRNA sequence^76^ into pLX-sgRNA (gift from Eric Lander and David Sabatini: Addgene plasmid # 50662) using the following primers: GTCGAGTGTGCTACTCAACTCGTTTTAGAGCTAGAAATAGCAA (forward) and GAGTTGAGTAGCACACTCGACGGTGTTTCGTCCTTTCC (reverse). Virus generation and cell infection were as described above. Twenty-four hours after infection, infected cells were selected with blasticidin. Simultaneously, Cas9 was induced with doxycycline. Cells were maintained in blasticidin and doxycycline for 7−14 days before assessing for knockout by western blot.

### Overexpression of SDHC-R72C

SDHCKO was generated as described in previous section. Bacterial stock of cDNA clone of *SDHC* (BC033626) was purchased from transomic technologies. SDHC-R72C mutation was made using site-directed mutagenesis kit from Invitrogen following the manufacturer’s protocol and constructs containing wild-type and mutated *SDHC* genes were recloned in PLVX mammalian expression vector using the following primers: CATCTGCCACTGTGGCACTGG (forward) and GACATCGCCATGGGAAGAG (reverse). Lentivirus particles containing these constructs were transduced in SDHCKO myeloma cell lines KMS11 and L363.

### 10-Nonyl acridine orange (NAO) staining

0.125 × 10^6^ cells were harvested. Cells were washed with PBS and incubated with NAO at a final concentration of 1 µM for 30 min at 37 °C following which they were subject to Annexin-APC/DAPI staining. MFI of stain was evaluated on live cells by flow cytometry.

### Colony-forming unit (CFU) assay

CFU assays were performed using the CytoSelect 96-Well In Vitro Tumor Sensitivity Assay (Soft Agar Colony Formation) from Cell Bio Labs, Inc. with modifications for quantitation. Cells were harvested and plated at 0.03 × 10^6^ cells per well according to the manufacturer’s instructions and incubated for 8 days. For quantification of viable cells, Nitro blue tetrazolium (NBT) (Sigma) was added on day 7 at a concentration of 1 mg per mL to each well and incubated for 24 h at 37°C prior to reading the plate. The plates were read using a BioTek Lionheart plate reader under Texas Red autofluorescence. Images taken for one well were stitched together using the Gen5 Image+ 3.04 software in the red channel with a linear blend fusion method. Six focal planes were condensed into minimum Z projections and subsequently analyzed using Gen5 Cellular Analysis.

### CellTiter-Glo assay

10,000 cells per 100 µL were plated in triplicates in a 96-well white flat bottom plate (Greiner CELLSTAR # 655083) with inhibitors (25 nM IACS-010759, 100 µM TTFA, 5 nM antimycin, 0.1 mM azide and 2.5 nM oligomycin) for 24 h. ATP levels were assessed 10 min after adding 100 µL of CellTiter-Glo Assay Reagent (Promega # G9248) to each well using a BioTek SYNERGY H1 microplate reader following the manufacturer’s instructions.

### Bioinformatics analysis

Differential expression was estimated between patients with and without t(11;14) translocation, identified from SeqFISH data from the same database. R package, DESeq2 was used to calculate differential gene expression and perform statistical analysis. Multiple hypothesis testing was performed using independent hypothesis weighting using the IHW R Package. Data are presented as log2 of fold-change in patients with t(11;14) vs. all other patients. All relevant clinical and genetics data were obtained from the MMRF CoMMpass study version 11a. Statistical significance (adjusted *p* value < 0.01) is highlighted for gene names in bold-italic font*.*

### Statistical analyses and reproducibility

Results are expressed as ±SEM of at least three independent experiments unless indicated otherwise. *p* values are indicated as follows: *p* < 0.0001 as **** and *p* > 0.05 as ns. Representative western blot images from one of three independent experiments are presented unless indicated otherwise.

### Reporting summary

Further information on research design is available in the Nature Research Reporting Summary linked to this article.

## Supplementary information

Supplementary Information

Peer Review File

Reporting Summary

## Data Availability

The CoMMpass MM trial (NCT0145429, IA11) data referenced during the study are available in a public repository from the MMRF (https://research.themmrf.org/) website. The source data underlying Figs. 1–7 and Supplementary Figs. 1−14 are provided as a Source Data file. All the other data supporting the findings of this study are available within the article and its supplementary information files and from the corresponding author upon reasonable request.

## Source Data

Source Data
