# Supplementary material for: Electron transport chain activity is a predictor and target for venetoclax sensitivity in multiple myeloma
Source: Nat Commun. 2020 Mar 6;11:1228. doi: 10.1038/s41467-020-15051-z (PMC7060223; doi:10.1038/s41467-020-15051-z)
Supplement: Supplementary file 1 — Supplementary Information [file 41467_2020_15051_MOESM1_ESM.pdf]

Supplementary Information

Electron transport chain activity is a predictor and target for venetoclax sensitivity in multiple myeloma

Bajpai and Sharma et al.

Supplementary Table 1: Characteristics of 50 myeloma patients tested for Ven +/- TTFA

Sample No.	Sample ID	Disease	ISS	karyotype	FISH	prior lines	Len ref	BZ ref	CZ ref	Pom ref	Dara ref	ASCT	Dex	Response to Venetoclax	IC50 of Ven (μM)	IC50 of Ven + TTFA (μM)
1	PS10001245-2	MM	ND	ND	t(11;14), +1q, -13	2	Y	N	N	N	N	Y	Y	PR	0.04009	0.006375
2	PS10001591	MM	2	complex	+1q, +11	0	N	N	N	N	N	N		CR	3.53E-09	3.01E-10
3	PS10001620	MM	ND	46XY	t(11;14), +9, -13, -17	1	N	N	N	N	N	Y	Y	sCR on car/ven	0.01673	0.000322
4	PS10001750	MM	3	complex	t(4;14), +1p, +1q, +3, +7, +9, +11, -13, del(17p)	1	Y	N	N	N	Y	Y			0.001328	0.00007358
5	PS10001677	MM	3	ND	+1q, del(13q)	4	Y	Y	Y	Y	Y	N			0.3915	0.03543
6	PS10001725	PCL	1	complex	-1p, +1q, +3, -13, -14, -17	5	N	N	Y	Y	Y	Y			0.1347	0.02717
7	PS10001731	MM	3	ND	ND	3	Y	N	N	Y	Y	Y			0.1525	0.03373
8	PS10001638	MM	ND	46XY	t(11;14)	1	Y	Y	N	N	N	N			0.3888	0.009763
9	PS10001643	MM	ND	46XY	t(11;14), +1p, +1q, +3, +7, +9, +13 del(17p)	6	Y	Y	Y	Y	Y	Y	Y	VGPR	0.3194	0.00207
10	PS10001651	MM	ND	46XX	-1p, +1q, +3, +9, +11, -14q	1	Y	N	N	N	N	Y			0.8008	2.06E-06
11	PS10001633	MM	2	46XX	negative	0	N	N	N	N	N	N			0.1709	0.006803
12	PS10001664	MM	3	46XX	+1q	2	N	N	N	N	N	Y			0.1391	0.02645
13	PS10001194	MM	1	ND	ND	4	Y	N	N	Y	Y	Y			0.115	0.001952
14	PS10001695	MM	ND	46XY	t(11;14)	3	Y	Y	N	N	N	N			1.512	0.01734
15	PS10001551-2	MM	ND	complex	t(11;14), +1q, +9, -13, del(17p)	2	Y	Y	N	N	N	N		post treatment sample: Ven relapse	0.955221	0.08115
16	PS10001619	MM	1	complex	+3, +7, +9, +11, +17	1	N	N	N	N	N	N			0.57	0.08235
17	PS10001658	MM	2	46XX	t(11;14), +1q, -13	2	Y	N	N	Y	N	Y	Y	SD	0.4284	0.07859

18	PS10001575	MM	ND	complex	t(11;14), +1p, +9, del(13q), del(17p)	9	Y	N	Y	Y	Y	Y		post treatment sample	0.906646	0.07954
19	PS10001683	MM	2	complex	+3, +9, +11q	1	Y	N	N	N	N	N			0.9219	0.06378
20	PS10001661-2	MM	2	46XY	-1p, +11, -13, +14	6	Y	N	Y	Y	Y	Y			1.36	0.1391
21	PS10001667	MM	3	46XY	t(11;14), del(13q)	1	Y	N	N	N	N	Y	N	sCR	0.1847	0.1426
22	PS10001674	MM	1	46XX	+1q, +9, +11q, del(13q)	5	Y	Y	Y	Y	Y	Y			0.609219	0.539597
23	PS10001570	MM	ND	46XX	t(11;14), +1, +3, +7, +9, +13, +17	3	N	Y	N	N	N	Y	N	sCR	0.5532	0.521725
24	PS10001581	MM	1	complex	+1, +3, +7, +9, +11	4	Y	Y	N	Y	Y	Y			2.875	1.669153
25	PS10001556	MM	ND	46XX	t(11;14)	3	Y	Y	N	N	N	N			3.636	1.63
26	PS10001406-2	MM	ND	46XX	t(11;14), +1q, -13	13	Y	Y	Y	Y	Y	Y	Y	PR	4.555868	1.449
27	PS10001625	MM	1	complex	-1p, +1q, +9, -13	0	N	N	N	N	N	N			4.084	1.661975
28	PS10001618	MM	1	46XY	+9, +11	0	N	N	N	N	N	N			0.894877	0.757167
29	PS10001646	MM	1	complex	+7, +9, +11, -17	0	N	N	N	N	N	N			6.949	3.134469
30	PS10001647	MM	ND	46XY	+1q, +3, +7, +9, +14	1	N	N	N	N	N	Y			8.979264	0.7126
31	PS10001650	MM	2	46XX	t(11;14), +3, +7, +9, del(13q), del(17p)	0	N	N	N	N	N	N			1.540164	0.6821
32	PS10001572	MM	2	ND	t(14;16)	0	N	N	N	N	N	N			3.820009	1.197
33	PS10001589	MM	3	46XX	+1q, -4p, +11q, -13, +14q, del(17p), +17	4	Y	N	Y	Y	Y	Y			5.658	3.243746
34	PS10001395-2	MM	2	complex	-1p, +9, +11q	1	N	N	N	N	N	Y			0.7444	0.747344
35	PS10001666	MM	1	46XY	+1q, -13, +IgH	0	N	N	N	N	N	N			5.847596	5.399
36	PS10001636	MM	2	46XX	+9, -13, +IgH	5	Y	Y	Y	Y	Y	Y			6.327	1.431
37	PS10001686	MM	ND	46XX	negative	2	N	N	N	N	N	N			4.883	2.47

38	PS10001684	MM	3	complex	+3, +9, +11q	0	N	N	N	N	N	N			0.6995	0.6854
39	PS10001699	MM	1	ND	+3, +7, +9, +11q	0	N	N	N	N	N	N			9.432763	6.956
40	PS10001555	PCL	2	46XX	t(11;14), +1, +3, del(13q), +17	9	Y	Y	Y	Y	Y	Y		post treatment (1/2018)	1.881508	1.530745
41	PS10001702	MM	3	complex	t(4;14), +1q, -1p, +3, +7, +11q	0	N	N	N	N	N	N			6.997025	1.12548
42	PS10001690-2	MM	1	complex	-1p, +1q, del(13q), +14q, del(17p)	3	Y	Y	Y	Y	Y	Y			0.681245	0.685949
43	PS10001669	MM	ND	46XX	t(11;14), +1q,-13	1	N	N	N	N	N	Y			10.345	9.432763
44	PS10001482	MM	2	ND	t(11;14), +1q, -3	7	Y	N	N	Y	Y	Y			0.3608	0.278
45	PS10001732	ND	ND	ND	ND	0	N	N	N	N	N	N			1.063	1.011
46	PS10001565	MM	2	complex	vt(11;14), +1, +3, +7, +9, +17p	3	Y	Y	N	Y	Y	Y			0.5404	2.343
47	PS10001653	MM	2	complex	-1p, +3, +7, +9, +11, +IgH	0	N	N	N	N	N	N			0.6231	0.4943
48	PS10001681	MM	1	ND	+3, +7, +9, +11, +17	0	N	N	N	N	N	N			1.330198	0.2383
49	PS10001622	MM	ND	46XY	+1q, +3, +7, +9, +11, -13, +14, del(17p), +17	4	Y	Y	Y	Y	Y	Y			3.461	0.2092
50	PS10001678	MM	1	ND	-1p, -13, +IgH, -17	0	N	N	N	N	N	N			0.306	0.4797

Supplementary Table 2: Characteristics of 9 myeloma patients tested for Ven +/- IACS

Sample No.	Sample ID	Disease	ISS	karyotype	FISH	prior lines	Len ref	BZ ref	Pom ref	Dara ref	ASCT	Dex	Response to Ven	IC50 of Ven (µM)	IC50 of Ven + IACS (µM)
1	PS10001243	MM	1	normal	+3, +7, +9, +11, +17	2	N	Y	N	N	N	N		ND	ND
2	PS10001818	MM	3	complex	del(1p), +3, +7, +9, +11, del(13q), del(17p)	0	N	N	N	N	N	N		21.48	0.6104
3	PS10001835	MM	NA	NA	+1q, +3, -13, +14	9	Y	N	Y	Y	Y	N		5.833	2.587
4	PS10001822	MM	NA	complex	+1q, +3, +7, +9, +11, del(13q)	0	N	N	N	N	N	N		3.318	0.3357
5	PS10001832	MM	1	NA	t(11;14), +1q, -13, -16	0	N	N	N	N	N	N		2.018	0.01732
6	PS10001837	MM	NA	normal	+1q, +3, +7, +9, -13, -14, +17	1	N	N	N	N	N	N		1.145	1.111
7	PS10001824	MM	1	normal	+1, +3, +7, +9, +11, +13, , +14, +17	0	N	N	N	N	N	N		0.1553	0.1392
8	PS10001834	MM	3	NA	+3, +9, +11, +17	0	N	N	N	N	N	N		0.03992	0.02831
9	PS10001582	MM	3	normal	t(11;14), +1q	3	Y	N	Y	Y	Y	Y	CR	0.009957	0.000209

* ND denotes IC50 values could not be determined.

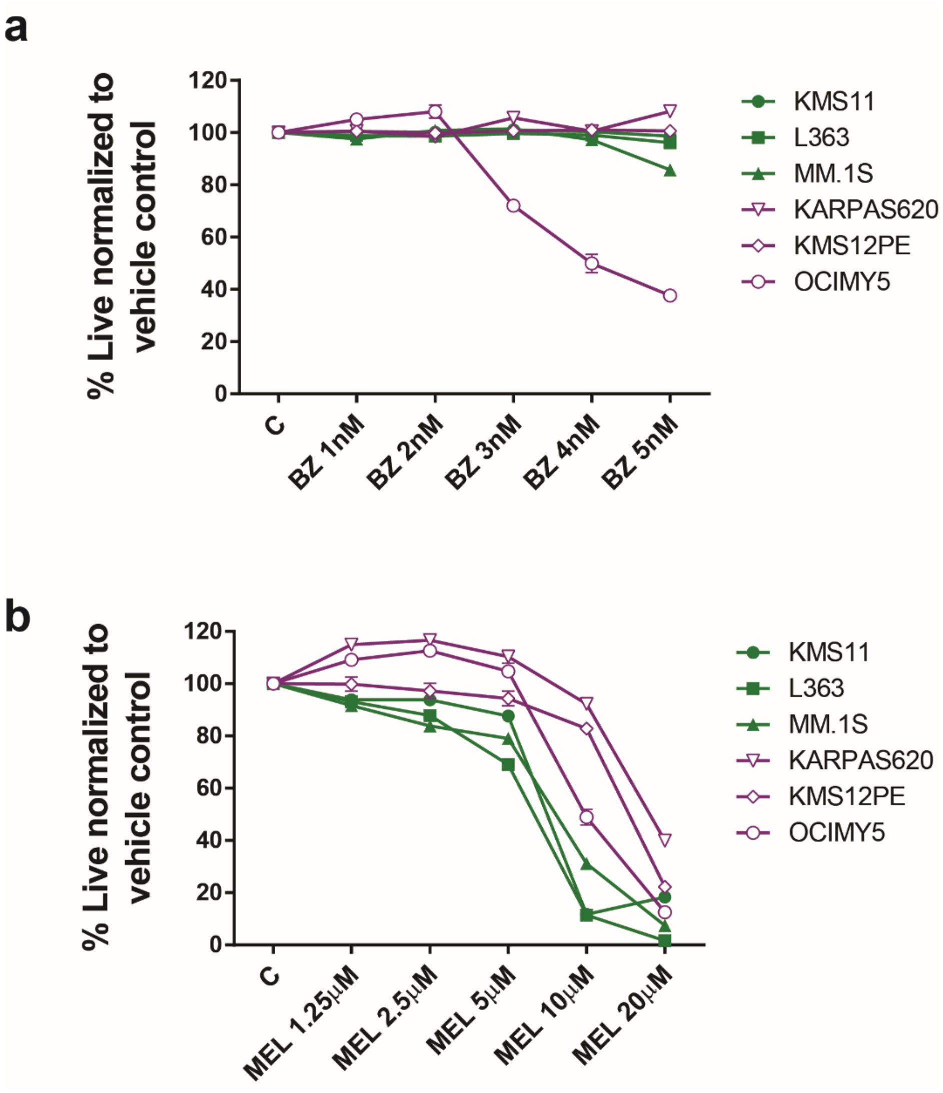

Supplementary Figure 1: Pattern of venetoclax sensitivity detected in MM lines is not represented in sensitivity to bortezomib or melphalan: Venetoclax resistant cell lines (KMS11, L363 and MM.1S) and venetoclax sensitive cell lines (KARPAS-620, KMS12PE and OCI-MY5) were cultured for 24 hrs in the presence of the indicated doses of **(a)** bortezomib (BZ: 1, 2, 3, 4 and 5nM) or **(b)** melphalan (MEL: 1.25, 2.5, 5, 10 and 20µM). Cell viability was measured by AnnexinV-DAPI staining and flow cytometry. n = 3 independent experiments. Data are presented as mean values +/- SEM. Source data are provided as a source data file.

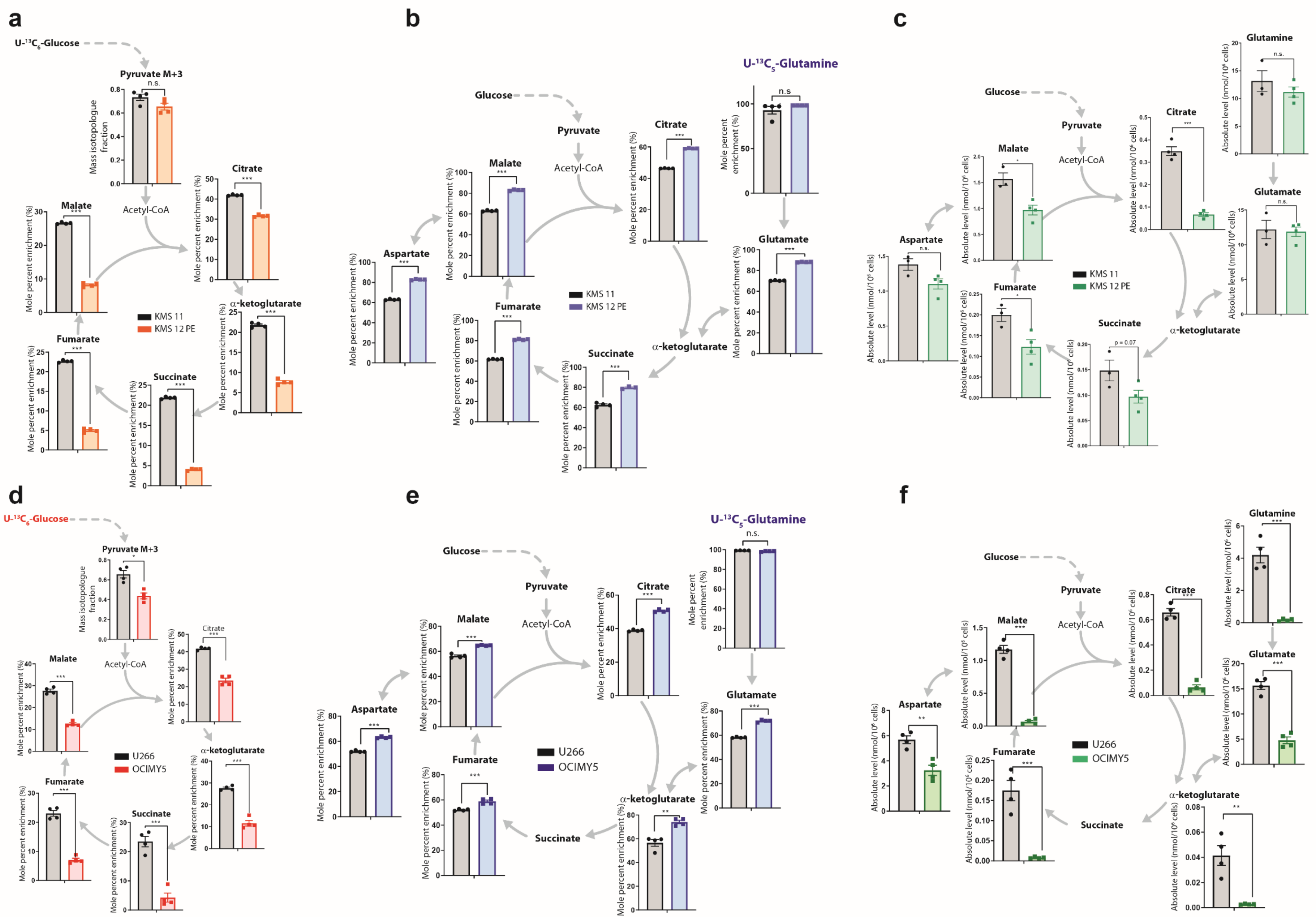
a
U-13C6-Glucose
Pyruvate M+3
n.s.
Mass isotopologue fraction
Acetyl-CoA
Citrate
Malate
Fumarate
Succinate
α-ketoglutarate
Mole percent enrichment (%)
KMS 11
KMS 12 PE
b
Glucose
Pyruvate
Acetyl-CoA
U-13C5-Glutamine
n.s
Citrate
Glutamate
Malate
Aspartate
Fumarate
Succinate
α-ketoglutarate
Mole percent enrichment (%)
KMS 11
KMS 12 PE
c
Glucose
Pyruvate
Acetyl-CoA
Glutamine
Glutamate
Citrate
Malate
Aspartate
Fumarate
Succinate
α-ketoglutarate
p = 0.07
Absolute level (nmol/10⁶ cells)
KMS 11
KMS 12 PE
d
U-13C6-Glucose
Pyruvate M+3
Mass isotopologue fraction
Acetyl-CoA
Citrate
Malate
Fumarate
Succinate
α-ketoglutarate
Mole percent enrichment (%)
U266
OCIMY5
e
Glucose
Pyruvate
Acetyl-CoA
U-13C5-Glutamine
n.s.
Citrate
Glutamate
Malate
Aspartate
Fumarate
Succinate
α-ketoglutarate
Mole percent enrichment (%)
U266
OCIMY5
f
Glucose
Pyruvate
Acetyl-CoA
Glutamine
Glutamate
Citrate
Malate
Aspartate
Fumarate
Succinate
α-ketoglutarate
Absolute level (nmol/10⁶ cells)
U266
OCIMY5

Supplementary Figure 2: Venetoclax sensitive cell lines show lower glucose incorporation and total levels of TCA cycle intermediates. Isotopologue labelling profiles and total levels of shown metabolites in **(a-c)** resistant non t(11;14) KMS11 and sensitive t(11;14) KMS12PE, and **(d-f)** resistant t(11;14) U266 and sensitive non t(11;14) OCIMY5 cells, supplemented with U-^{13}C-glucose or U-^{13}C-glutamine for 24 hrs. n = 4 independent experiments. Data are presented as mean values +/- SEM. p-values are calculated using two-tailed t-tests with Welch's correction. Source data are provided as a source data file.

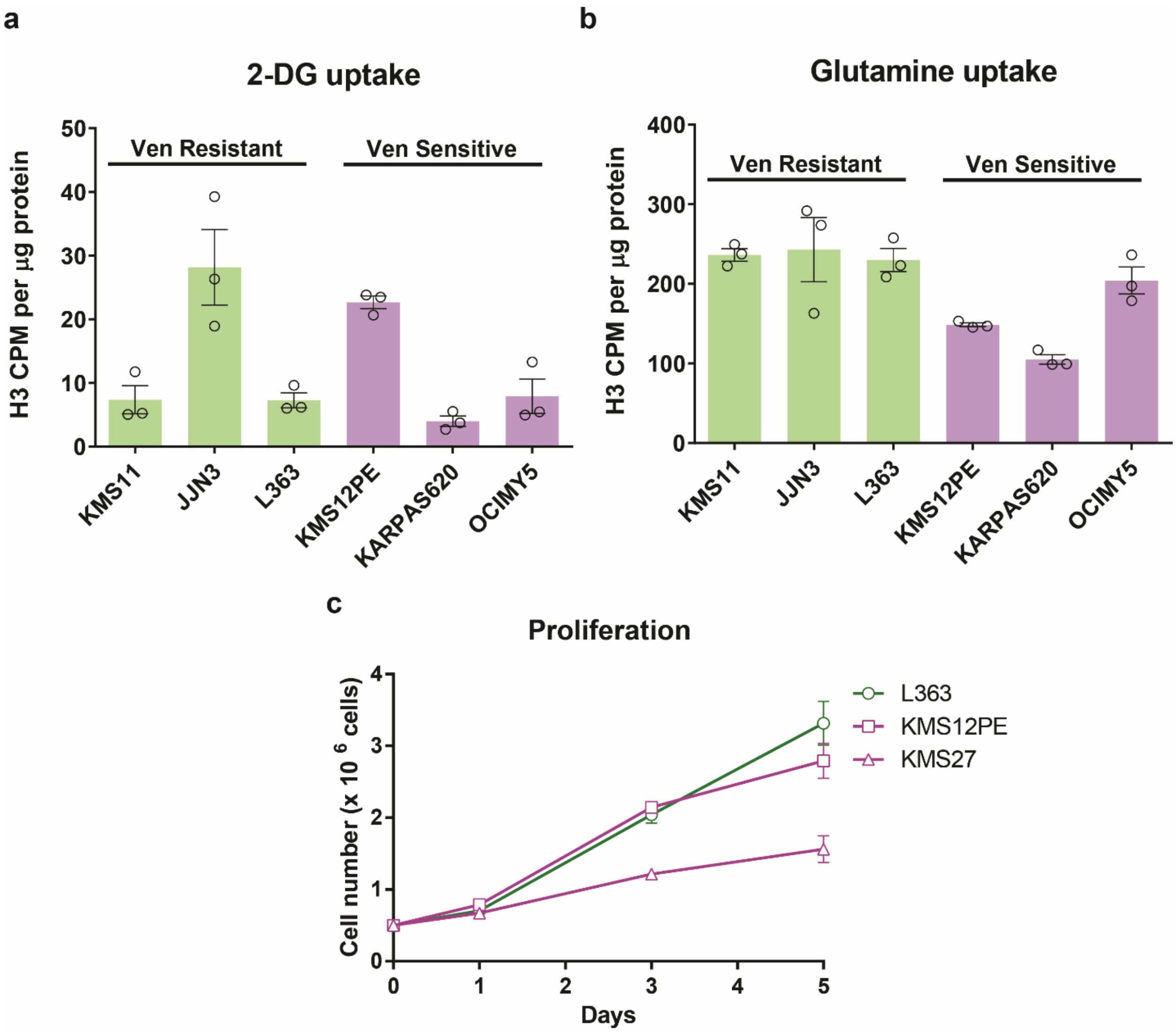

Supplementary Figure 3: [3H]-2-deoxyglucose or [3H]-glutamine uptake and proliferation rates of MM lines do not correlate with sensitivity to venetoclax: (a-b) 1 x 10^6 cells each from venetoclax resistant cell lines (KMS11, JJN3 and L363) , shown in green, and venetoclax sensitive cell lines (KMS12PE, KARPAS-620 and OCI-MY5) , shown in purple, were plated in polyethylamine (PEE) (25µg per mL PEE and 0.15M NaCl in water) pre-coated 6-well plates and starved of either **(a)** glucose or **(b)** glutamine for 30min. 0.05mM cold 2-deoxyglucose and 0.05mM [3H]-2-deoxyglucose were added for 6min in (a), and 0.05mM cold glutamine and 0.05mM [3H]-glutamine were added for 15min in (b). Cells were washed, lysed and equal amounts of protein were evaluated by scintillation counting. n = 3 independent experiments. Data are presented as mean values +/- SEM. **(c)** L363, KMS12PE and KMS27 cell lines were cultured in media for 5 days. Cell counting was performed using BIORAD cell counter at 0, 1st, 2nd and 5th day. Source data are provided as a source data file. n = 3 independent experiments. Data are presented as mean values +/- SEM.

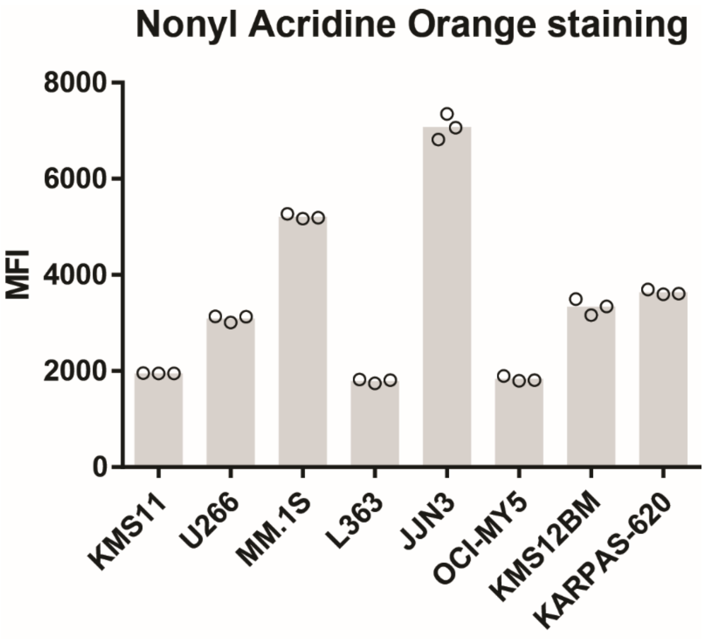

Supplementary Figure 4: Mitochondrial mass does not differentiate venetoclax sensitive and resistant MM: Venetoclax resistant cell lines (KMS11, U266, MM.1S, L363 and JJN3) and venetoclax sensitive cell lines (OCI-MY5, KMS12BM and KARPAS-620) were stained with nonyl acridine orange (NAO) dye for 30min. NAO binds to cardiolipin in the inner mitochondrial membrane regardless of the energetic state and can be used as an indirect correlate of mitochondrial content. Median fluorescence intensity (MFI) was measured by flow cytometry at an excitation of 495nm and emission of 519nm. n = 3 technical replicates. Source data are provided as a source data file.

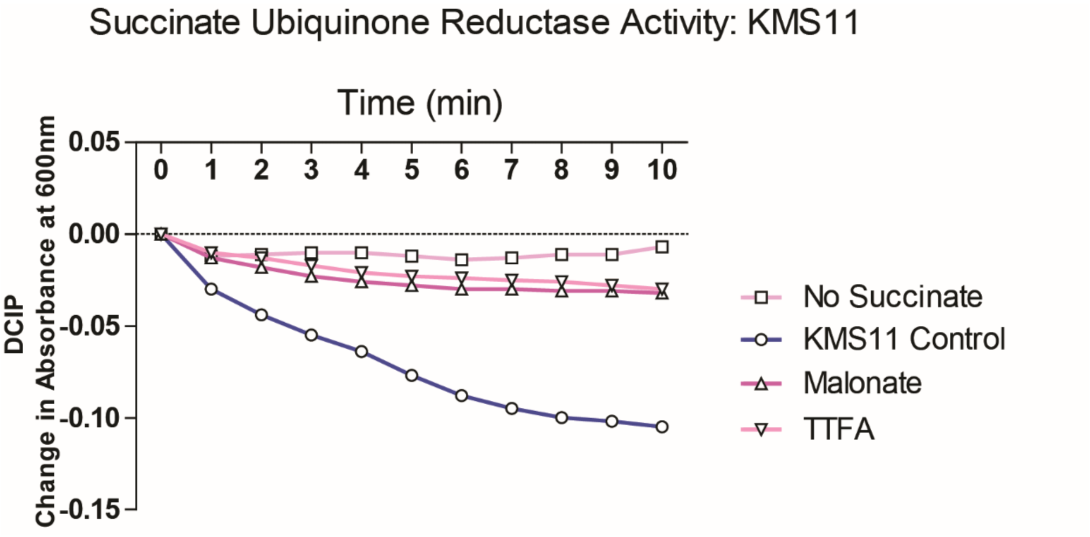

Supplementary Figure 5: TTFA, malonate or lack of succinate inhibit succinate ubiquinone reductase activity in a SQR assay: SQR activity was performed in KMS11 cells (n = 1 well per condition) as described in methods. Absence of succinate, or presence of TTFA or malonate do not change the absorbance of DCPIP (600nm) in contrast to the control cells, reflecting reduced SQR activity. Source data are provided as a source data file.

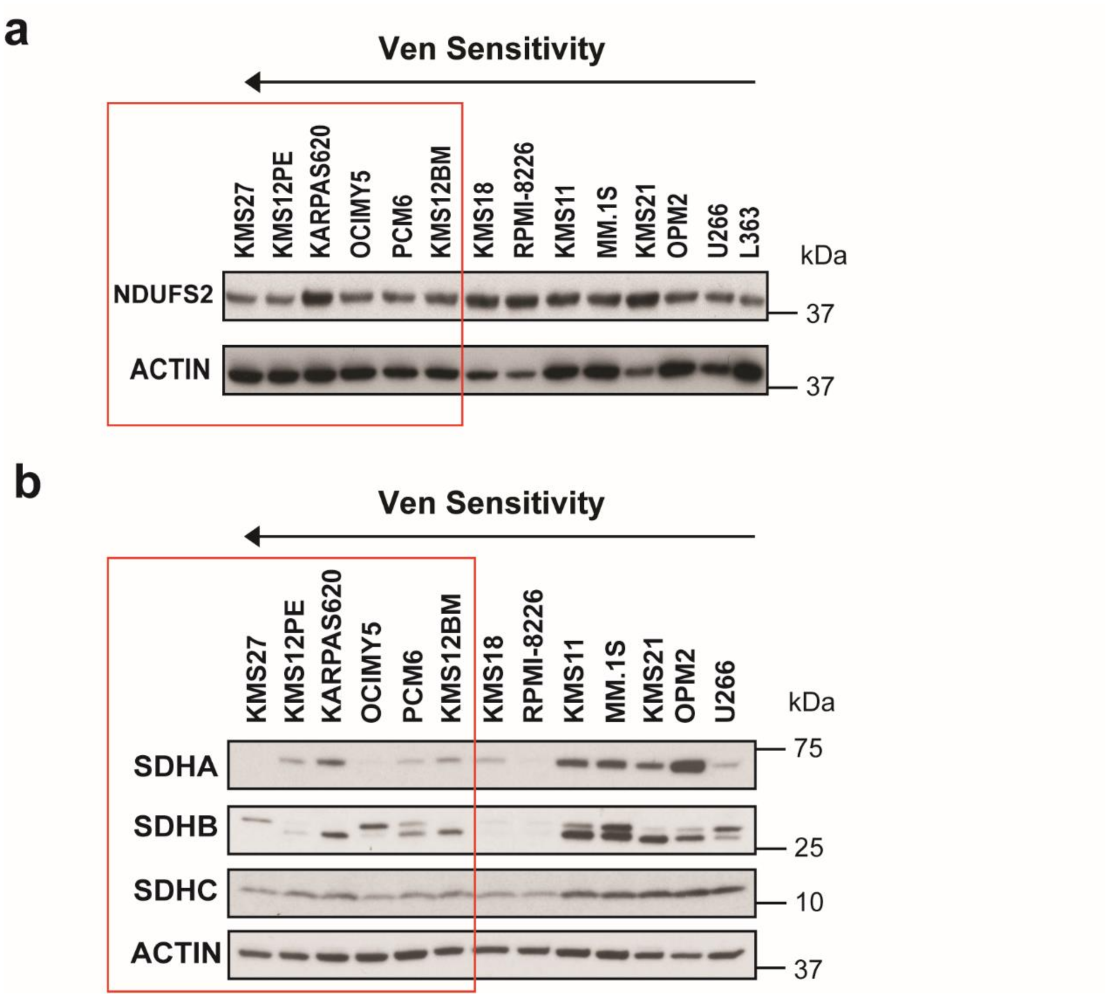

Supplementary Figure 6: Complex I subunit NDUFS2 and SDHA, B and C subunit expression levels do not correlate with Complex I or SDH/SQR activity respectivly: (a) Lysates from venetoclax resistant cells (KMS18, RPMI-8226, KMS11, MM.1S, KMS21BM, OPM2, U266 and L363) and venetoclax sensitive cell lines (KMS27, KMS12PE, KARPAS-620, OCI-MY5, PCM6 and KMS12BM) were prepared and expression of Complex I subunit NDUFS2 and actin as a loading control were evaluated by western blotting. Representative blots from one of two independent experiments is presented. **(b)** Lysates from venetoclax resistant cells (KMS18, RPMI-8226, KMS11, MM.1S, KMS21BM, OPM2 and U266) and venetoclax sensitive cell lines (KMS27, KMS12PE, KARPAS-620, OCI-MY5, PCM6 and KMS12BM) were prepared and expression of the indicated SDH subunits and actin as a loading control were evaluated by western blotting. Source data are provided as a source data file.

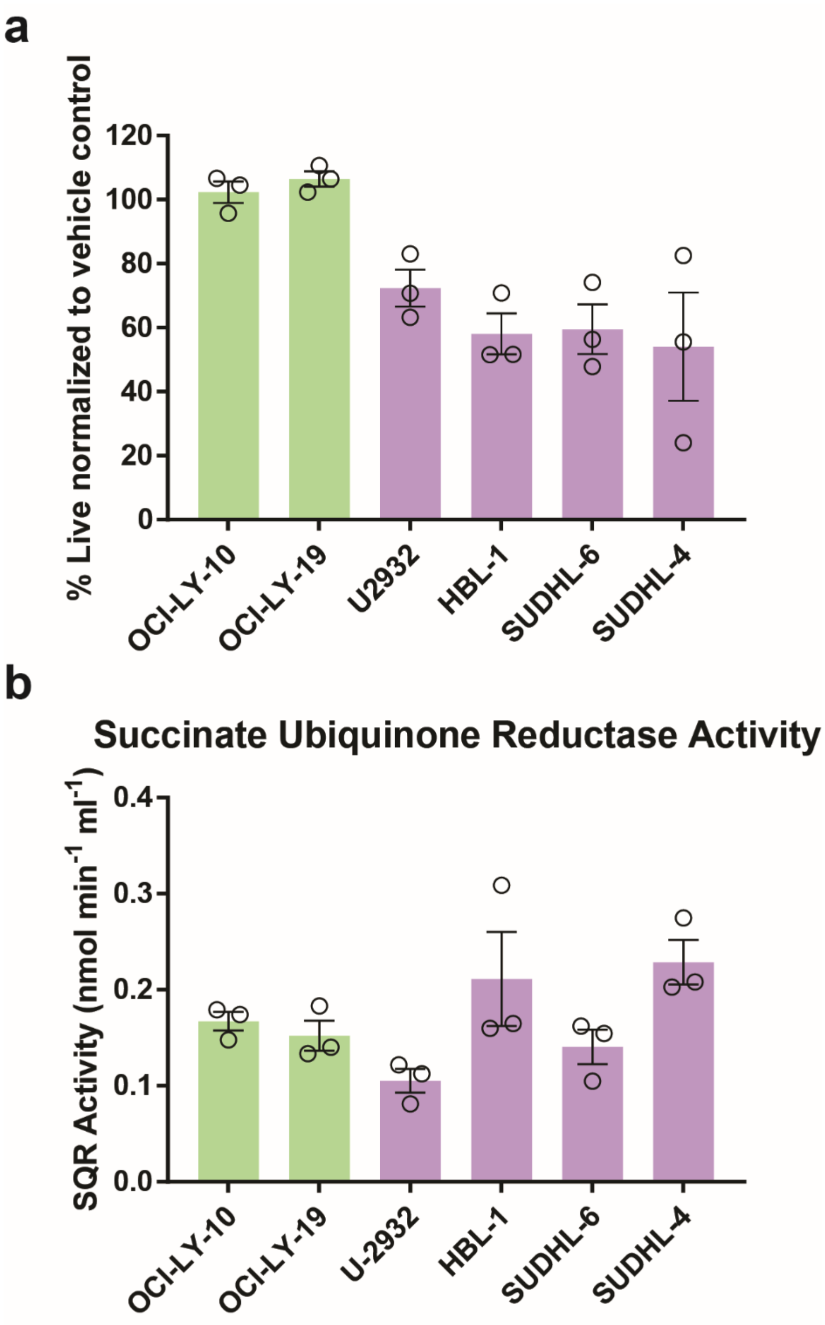

Supplementary Figure 7: SQR activity of DLBCL lines does not correlate with venetoclax sensitivity: (a) DLBCL cell lines treated with 0.5µM venetoclax for 24 hrs, were assessed for cell death by AnnexinV/DAPI flow cytometric staining. Percent live normalized to vehicle control with cell lines grouped by sensitivity (Venetoclax resistant lines in green and venetoclax sensitive lines in purple). n = 3 independent experiments. Data are presented as mean values +/- SEM. **(b)** SQR activity was assessed in gently permeabilized whole cells supplemented with succinate and Complex I, III, and IV inhibitors. n = 3 independent experiments. Data are presented as mean values +/- SEM. Source data are provided as a source data file.

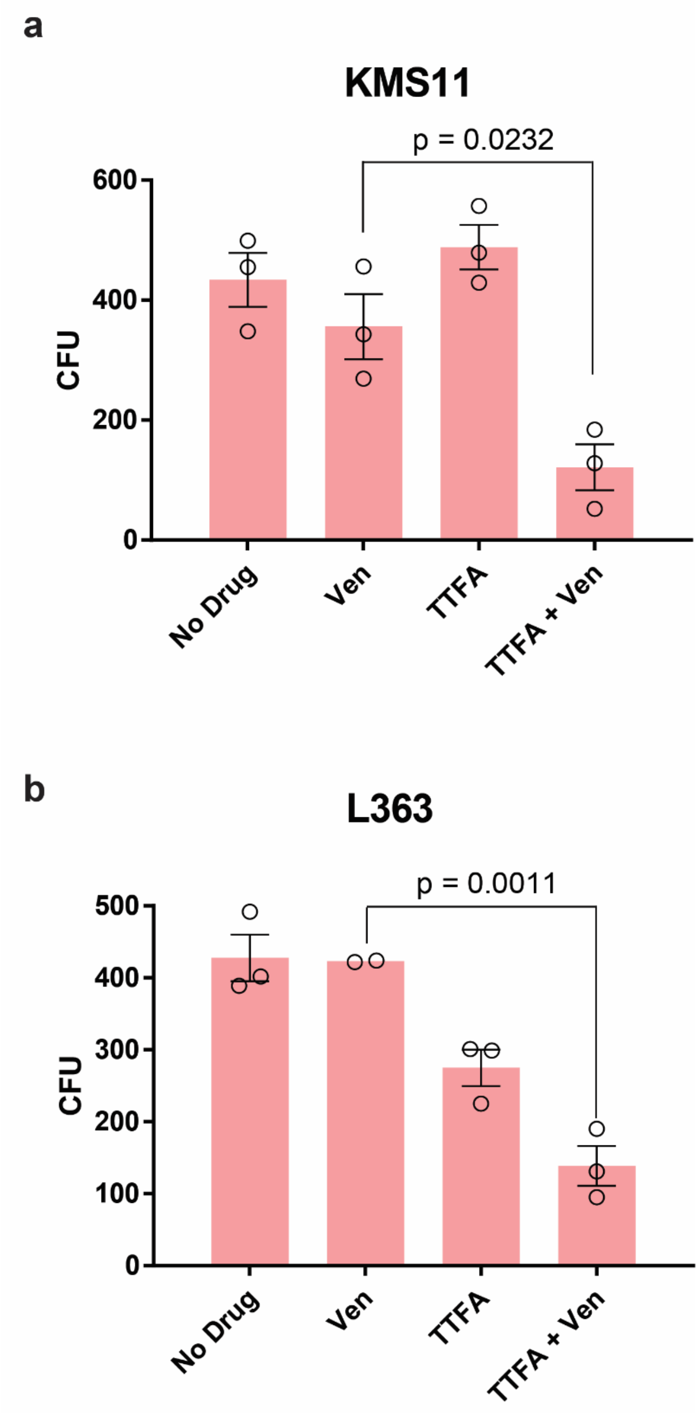

Supplementary Figure 8: TTFA sensitizes L363 and KMS11 cells to venetoclax in a colony forming assay. (a) KMS11 and **(b)** L363 were treated with venetoclax (0.1µM) and TTFA (100µM) or the combination in soft agar in a 96-well plate for 7 days and the number of colonies was assessed using NBT dye under Texas Red autofluorescence. n = 3 independent experiments. Data are presented as mean values +/- SEM. Adjusted p-values are calculated using a one-way ANOVA with post-hoc Tukey's multiple comparisons test. Source data are provided as a source data file.

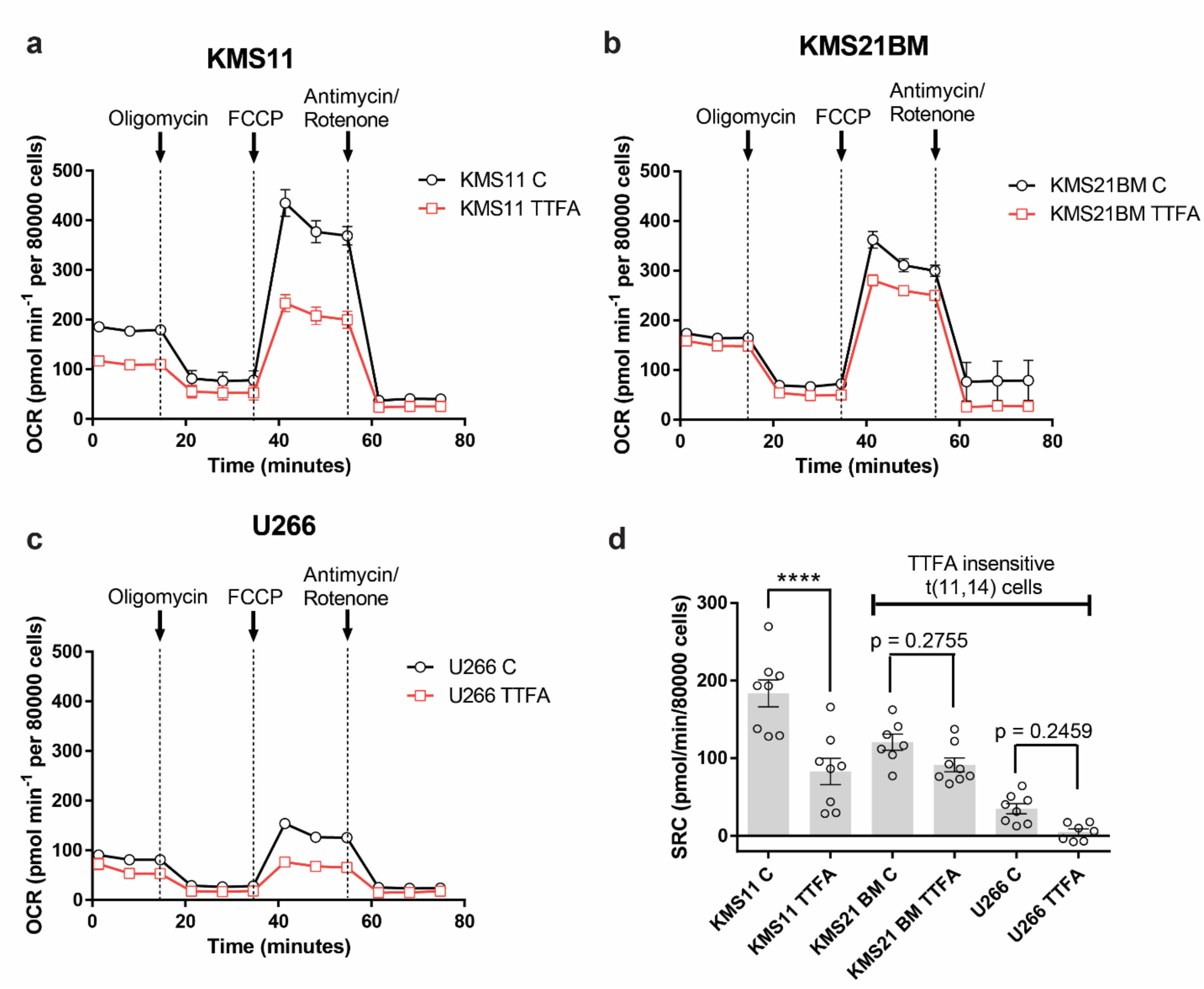

Supplementary Figure 9: Investigation of OCR in TTFA + Ven resistant t(11;14) MM cells. Oxygen consumption rate (OCR) in **(a)** KMS11, **(b)** KMS21BM, and **(c)** U266 determined after treating cells with or without 100µM TTFA for 24 hrs. n = 8 Seahorse wells except for KMS21BM C and U266 TTFA (n = 7). Data are presented as mean values +/- SEM. **(d)** Spare respiratory capacity (SRC) derived from (a-c) . Data are presented as mean values +/- SEM. Adjusted p-values are calculated using a two-way ANOVA with post-hoc Sidak's multiple comparisons test. Source data are provided as a source data file.

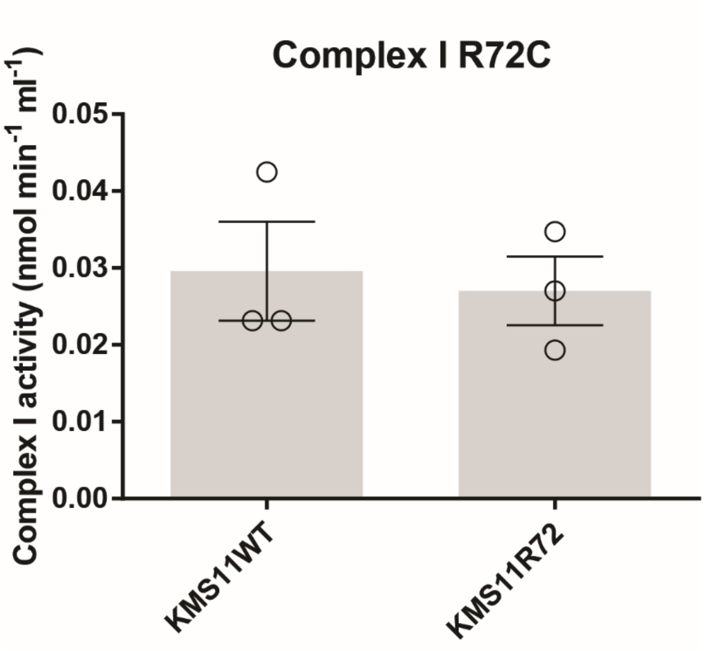

Supplementary Figure 10: SDHC-R72C mutant-expressing KMS11 cells exhibit intact Complex I activity: Complex I activity was assessed (as described in materials and methods) in KMS11 cells expressing the SDHC-R72C mutant or wild type SDHC. n = 3 independent experiments. Data are presented as mean values +/- SEM. Source data are provided as a source data file.

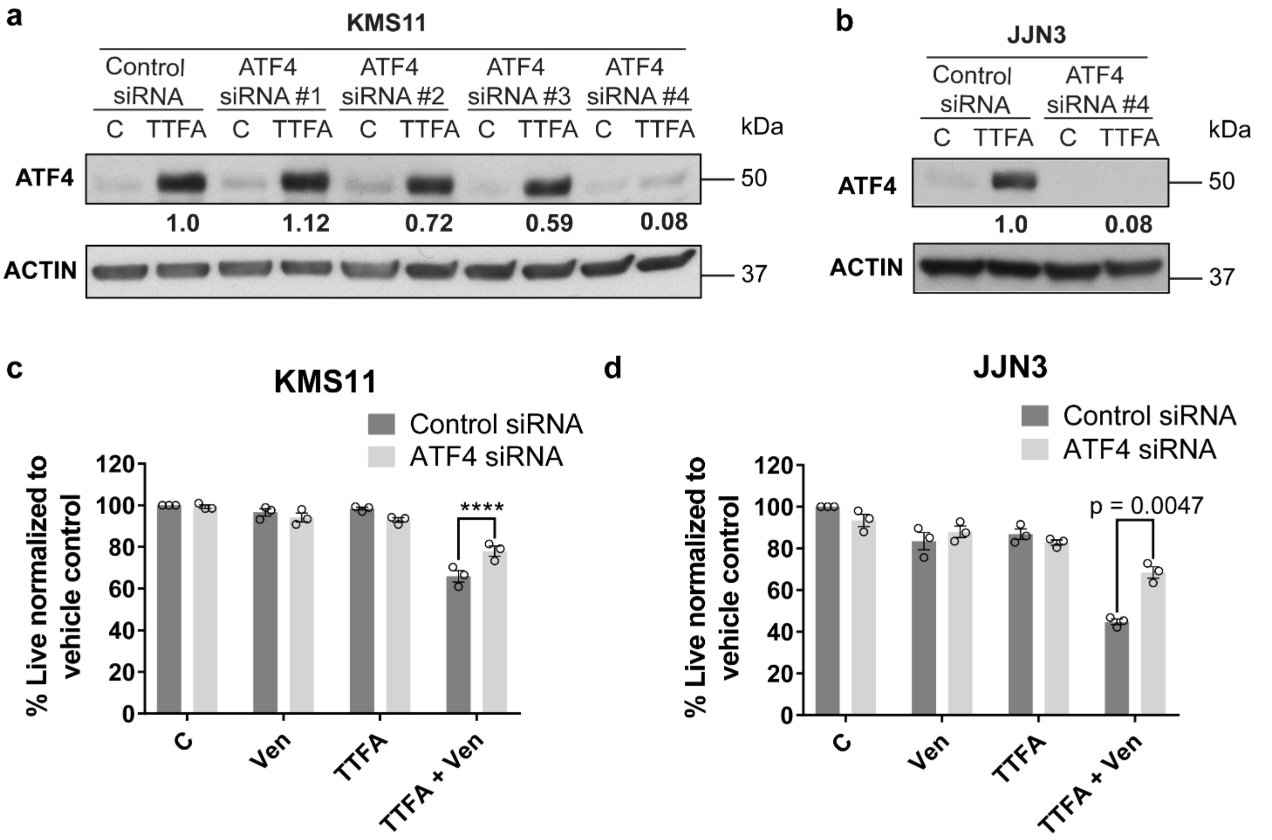

Supplementary Figure 11: Knockdown of ATF4 in KMS11 and JJN3 cells using individual siRNA reverses sensitivity to venetoclax: (a and b) KMS11 and JJN3 cells transfected with Control siRNA or ATF4 siRNA were treated with TTFA (100 µM) for 24 hrs and evaluated for expression of the indicated proteins by immunoblot analysis. Representative blots from one of three independent experiments is presented. **(c and d)** Transfected cells were treated with venetoclax (0.5 µM), TTFA (100 µM) or the combination for 24 hrs and cell viability was assessed by AnnexinV/DAPI flow cytometric staining. n = 3 independent experiments. Data are presented as mean values +/- SEM. Adjusted p-values are calculated using a two-way ANOVA with post-hoc Tukey's multiple comparisons test. Source data are provided as a source data file.

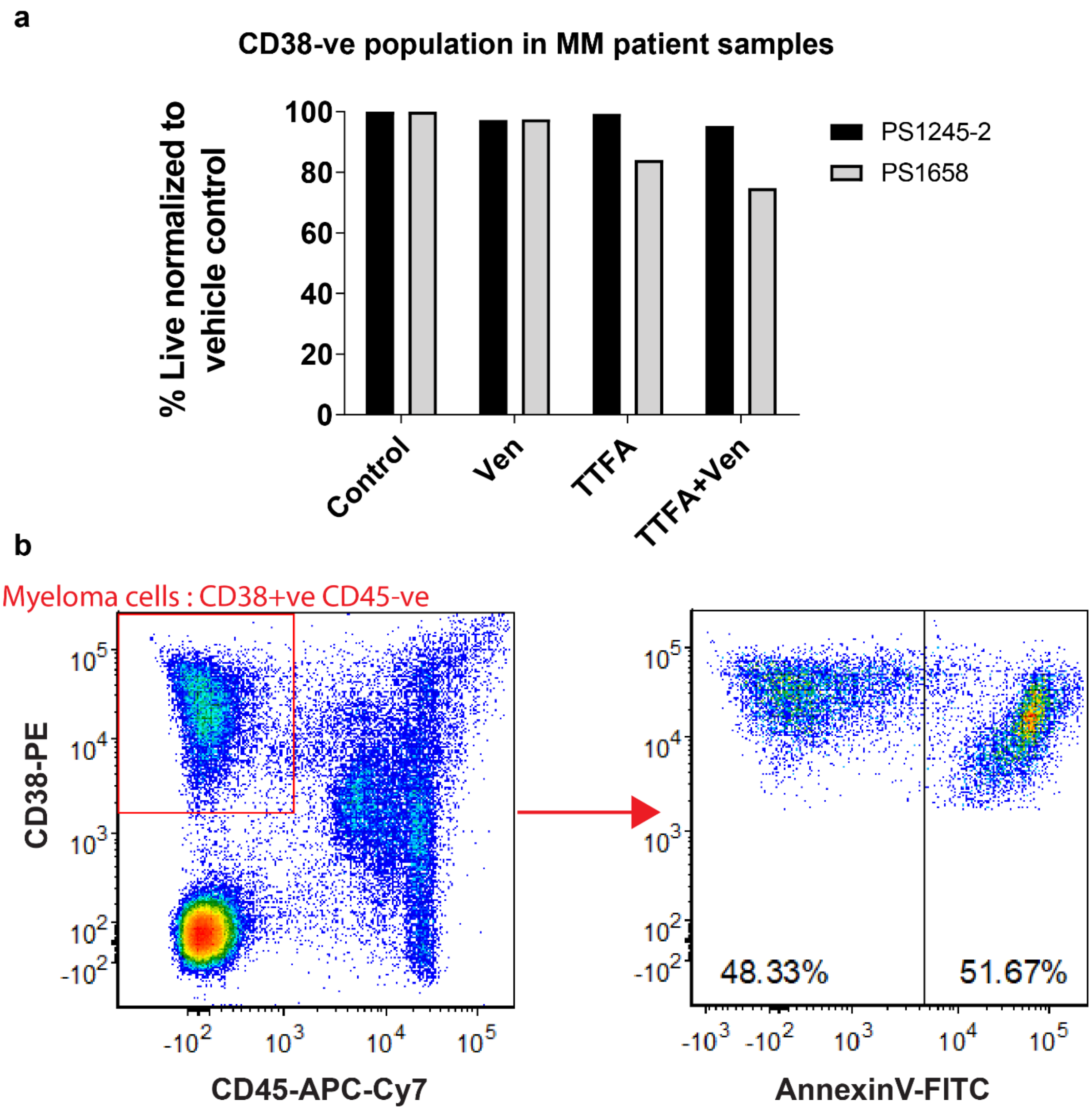

Supplementary Figure 12: Venetoclax or TTFA do not impact the viability of non-myeloma normal cellular populations contained in patient bone marrow aspirates: (a) Cells gated on CD38 -ve population were analyzed in PS10001245-2, a venetoclax sensitive patient sample bone marrow aspirate and PS10001658, a venetoclax resistant patient sample bone marrow aspirate. Aspirates were treated with venetoclax (0.1µM), TTFA (100µM) or the combination as indicated. Cell viability was assessed by AnnexinV staining using flow cytometry. Source data are provided as a source data file**. (b)** Gating strategy for analyzing patient samples: CD38-PE positive and CD45-APC-Cy7 negative cells were used to gate myeloma cells. Next, the gate was applied to the CD38-PE and Annexin-FITC flow plot to get percentage AnnexinV positive and negative myeloma cells.

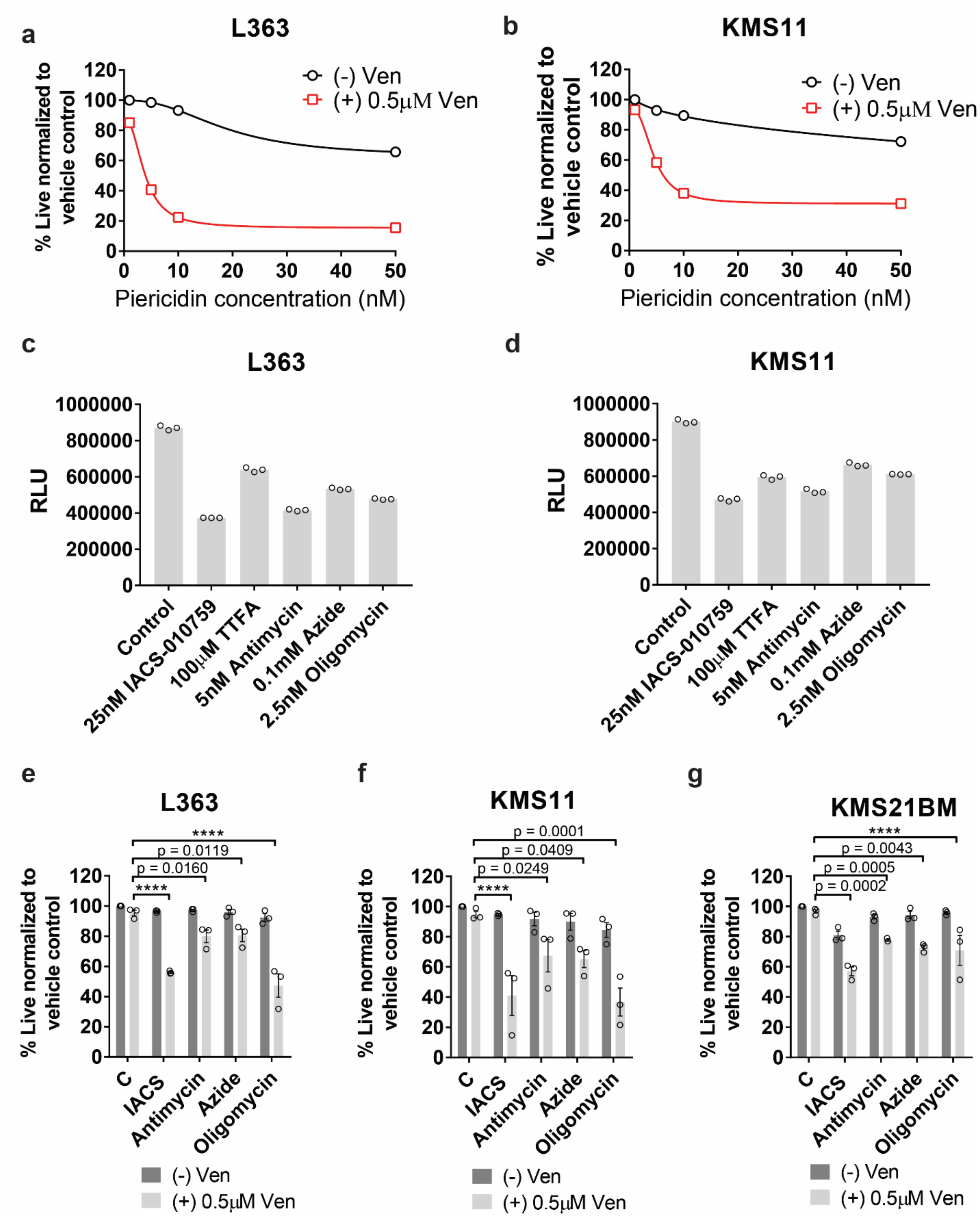

Supplementary Figure 13: Inhibition of Complex III, Complex IV or Complex V uniformly sensitizes resistant MM to Venetoclax: (a-b) Dose response curves for co-treatment of L363 and KMS11 with 0.5µM venetoclax and increasing doses of Complex I inhibitor piericidin for 24 hrs. Cell viability assessed by AnnexinV/DAPI staining. **(c-d)** Relative luminescence units indicative

of ATP levels in L363 and KMS11 on treatment with low doses of ETC inhibitors for 24 hrs. n = 3 technical replicates. **(e-g)** Complex I, Complex III, Complex IV or Complex V inhibition with IACS-010759 (25nM), antimycin (5nM), sodium azide (0.1mM) or oligomycin (2.5nM), respectively, sensitizes the indicated myeloma cell lines to 0.5μM of venetoclax on treatment for 24 hrs. Cell viability assessed by AnnexinV/DAPI staining. n = 3 independent experiments. Data are presented as mean values +/- SEM. Adjusted p-values are calculated using a two-way ANOVA with post-hoc Dunnett's multiple comparisons test. Source data are provided as a source data file.

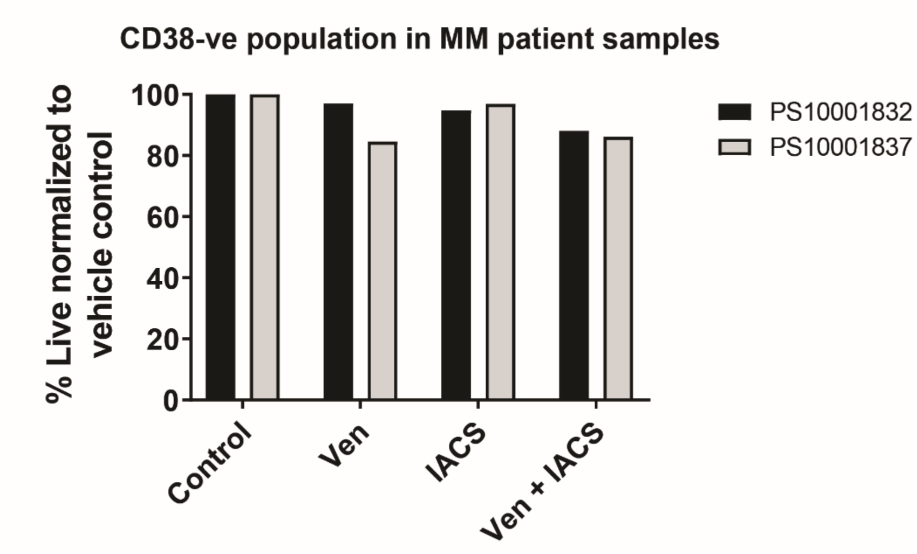

Supplementary Figure 14: IACS-010759 in combination with venetoclax does not impact the viability of non-myeloma normal cellular populations contained in patient bone marrow aspirates: Cells gated on CD38 -ve population were analyzed in PS10001832, and PS10001837. Aspirates were treated with the venetoclax (0.1µM) or IACS-010759 (25nM) or the combination as indicated. Cell viability was assessed by AnnexinV staining using flow cytometry. Source data are provided as a source data file.
