# Supplementary material for: Electron transport chain activity is a predictor and target for venetoclax sensitivity in multiple myeloma
Source: Nat Commun. 2020 Mar 6;11:1228. doi: 10.1038/s41467-020-15051-z (PMC7060223; doi:10.1038/s41467-020-15051-z)
Supplement: Supplementary file 3 — Reporting Summary [file 41467_2020_15051_MOESM3_ESM.pdf]

nature research

Corresponding author(s): Mala Shanmugam

Last updated by author(s): Jan 24, 2020

Reporting Summary

Nature Research wishes to improve the reproducibility of the work that we publish. This form provides structure for consistency and transparency in reporting. For further information on Nature Research policies, see Authors & Referees and the Editorial Policy Checklist.

Statistics

For all statistical analyses, confirm that the following items are present in the figure legend, table legend, main text, or Methods section.

n/a	Confirmed	
☐	☒	The exact sample size (*n*) for each experimental group/condition, given as a discrete number and unit of measurement
☐	☒	A statement on whether measurements were taken from distinct samples or whether the same sample was measured repeatedly
☐	☒	The statistical test(s) used AND whether they are one- or two-sided<br>*Only common tests should be described solely by name; describe more complex techniques in the Methods section.*
☒	☐	A description of all covariates tested
☐	☒	A description of any assumptions or corrections, such as tests of normality and adjustment for multiple comparisons
☐	☒	A full description of the statistical parameters including central tendency (e.g. means) or other basic estimates (e.g. regression coefficient) AND variation (e.g. standard deviation) or associated estimates of uncertainty (e.g. confidence intervals)
☐	☒	For null hypothesis testing, the test statistic (e.g. *F*, *t*, *r*) with confidence intervals, effect sizes, degrees of freedom and *P* value noted<br>*Give P values as exact values whenever suitable.*
☒	☐	For Bayesian analysis, information on the choice of priors and Markov chain Monte Carlo settings
☐	☒	For hierarchical and complex designs, identification of the appropriate level for tests and full reporting of outcomes
☐	☒	Estimates of effect sizes (e.g. Cohen's *d*, Pearson's *r*), indicating how they were calculated

Our web collection on statistics for biologists contains articles on many of the points above.

Software and code

Policy information about availability of computer code

Data collection	All relevant clinical and genetics data were obtained from the MMRF CoMMpass study version 11a.
Data analysis	R 3.5.3 package, DESeq2 was used to calculate differential gene expression and perform statistical analysis. Multiple hypothesis testing was performed using independent hypothesis weighting using the IHW R Package; XFe wave software (Seahorse Bioscience Inc., MA) for Seahorse data analysis; flow cytometric analysis done using De Novo Software FCS Express Version 4 and statistical analysis using Graph Pad Prism 7.04; Gen5 Image+ 3.04 software was used to do image analysis for the colony forming assay. http://crispr.mit.edu/ and http://www.broadinstitute.org/rnai/public/analysis-tools/sgrna-design-v1 web tools were used to design sgRNAs.

For manuscripts utilizing custom algorithms or software that are central to the research but not yet described in published literature, software must be made available to editors/reviewers. We strongly encourage code deposition in a community repository (e.g. GitHub). See the Nature Research guidelines for submitting code & software for further information.

Data

Policy information about availability of data

All manuscripts must include a data availability statement. This statement should provide the following information, where applicable:

- Accession codes, unique identifiers, or web links for publicly available datasets
- A list of figures that have associated raw data
- A description of any restrictions on data availability

CoMMpass data can be accessed at https://research.themmrf.org/ ; For in vitro and ex vivo studies sample sizes were determined based on our experience, published studies and availability of patient samples. No statistics was used to predetermine the sample size.

October 2018

Field-specific reporting

Please select the one below that is the best fit for your research. If you are not sure, read the appropriate sections before making your selection.

☒ Life sciences ☐ Behavioural & social sciences ☐ Ecological, evolutionary & environmental sciences

For a reference copy of the document with all sections, see nature.com/documents/nr-reporting-summary-flat.pdf

Life sciences study design

All studies must disclose on these points even when the disclosure is negative.

Sample size	The CoMMpass study size was determined as 1,000 newly diagnosed patients prior to study initiation based on power analyses to analyze gene expression within subtypes of myeloma. The CoMMpass study is an initiative of the MMRF.
Data exclusions	No data were excluded.
Replication	Information about the number of independent experimental repeats and technical replicates is provided in the paper for each experiment. All attempts at replication were successful. Experiments involving patient samples was done once.
Randomization	Cell lines and patient samples are defined by their unique genotypes. Therefore, no sample randomization was performed.
Blinding	Samples were not blinded as the defining characteristic i.e. sensitivity/resistance to venetoclax are what defined subsequent analyses.

Reporting for specific materials, systems and methods

We require information from authors about some types of materials, experimental systems and methods used in many studies. Here, indicate whether each material, system or method listed is relevant to your study. If you are not sure if a list item applies to your research, read the appropriate section before selecting a response.

Materials & experimental systems

n/a	Involved in the study	
☐	☒	Antibodies
☐	☒	Eukaryotic cell lines
☒	☐	Palaeontology
☒	☐	Animals and other organisms
☐	☒	Human research participants
☐	☒	Clinical data

Methods

n/a	Involved in the study	
☒	☐	ChIP-seq
☐	☒	Flow cytometry
☒	☐	MRI-based neuroimaging

Antibodies

Antibodies used	BCL-xL (54H6) (#2764S), BIM (C34C5) (#2933), PUMA (#4976), BAX (#2772), BID (#2002) and BCL-2 (D55G8) (#4223) antibodies were obtained from Cell Signaling Technology ; MCL-1 (S-19) (#sc-819)), SDHA (F-2) (sc-390381), SDHB (G-10) (#sc-271548), SDHC (C-2) (#sc-515102) and NDUFS2 (B-3) (#sc-390596) antibodies from Santa Cruz Biotechnology; ATF4 (#ab23760) antibody from Abcam; beta-actin (AC-15)(#A5441), BAK (#06-536) and NOXA (114C307) (#OP180) antibodies from Sigma-Aldrich. Antibody to BCL-2 (6C8) (Hamster) (#51-1513GR) antibody used in co-IP was purchased from BD-Pharmingen. All antibodies were used at a 1:1000 dilution.
Validation	All antibodies have been validated by the manufacturer for detection of human proteins by western blotting. BCL-xL (54H6) (#2764S) and BIM (C34C5) (#2933) were validated by CST using BCL-xl and BIM siRNA respectively. BAX (#2772) and BCL-2 (D55G8) (#4223) antibodies were validated by CST using BAX and BCL-2 knockouts. ATF4 (#ab23760) antibody was validated by Abcam using an ATF4 tagged recombinant protein. The BIM, SDHC and NOXA antibodies were further validated by us using the BIM, SDHC and NOXA KO lines.

Eukaryotic cell lines

Policy information about cell lines

Cell line source(s)	KMS18 (Dr. L Bergsagel, Mayo Clinic, AZ), RPMI-8226 (ATCC), U266, L363, KMS11, JJN3 (Dr. M Kuehl, NCI, MD), MM.1S (Dr. S Rosen, City of Hope, CA). KMS18, KMS21BM, KMS12BM, KMS12PE and KMS27 were obtained from Japanese Collection of Research Biosources. KARPAS-620, OCI-MY5 and H1112 were obtained from Jonathan Keats, TGEN, City of Hope affiliate. L363 and KMS11 Crispr/Cas9 BIM and SDHC knock out (KO) lines were generated in the Shanmugam lab and KMS18 and RPMI-8226 NOXA KO lines were generated in the Boise lab at Emory University, Atlanta, GA. DLBCL cell lines were obtained

	from the following sources: OCI-LY-19, OCI-LY-10 and HBL-1 from Dr. C Henry; SUDHL-4, SUDHL-6 and U2932 from Dr. L Mizrachi-Bernal, Emory University, Atlanta, GA. HEK293T were purchased from American Type Culture Collection.
Authentication	L363, KMS11, JJN3, MM.1S, KMS18, KMS12BM, KMS12PE and KMS27 were authenticated using Short Tandem Repeat (STR) DNA profiling by Emory Integrated Genomics Core. The other cell lines have been authenticated by the source.
Mycoplasma contamination	All cell lines tested negative for mycoplasma
Commonly misidentified lines (See ICLAC register)	KMS21BM

Human research participants

Policy information about studies involving human research participants

Population characteristics	Patients of any age or sex with lymphoid cancers diagnosed in the state of Georgia.
Recruitment	Patients living in Georgia or traveling to Georgia at least occasionally for medical standard of care and/or follow-up treatment can donate to the Myeloma/Lymphoma Sample Repository.
Ethics oversight	Emory University Institutional Review Board

Note that full information on the approval of the study protocol must also be provided in the manuscript.

Clinical data

Policy information about clinical studies

All manuscripts should comply with the ICMJE guidelines for publication of clinical research and a completed CONSORT checklist must be included with all submissions.

Clinical trial registration	NCT0145429
Study protocol	CoMMpass patients were all newly diagnosed with multiple myeloma and were recruited from North American and European collection sites. Patients were treated with physician's choice of regimens but had to include either a 1) proteasome inhibitor or 2) IMiD based regimen.
Data collection	Patients were recruited by treating physicians through the MMRC.
Outcomes	Ongoing

Flow Cytometry

Plots

Confirm that:

- [x] The axis labels state the marker and fluorochrome used (e.g. CD4-FITC).
- [x] The axis scales are clearly visible. Include numbers along axes only for bottom left plot of group (a 'group' is an analysis of identical markers).
- [x] All plots are contour plots with outliers or pseudocolor plots.
- [x] A numerical value for number of cells or percentage (with statistics) is provided.

Methodology

Sample preparation	Bone marrow aspirates or peripheral blood samples from consenting myeloma patients were diluted to 25 mL with 1X PBS and over laid on lymphocyte separation media (Corning). Following centrifugation, the collected buffy coat was washed with PBS and re-suspended in culture medium. Cells subject to various treatments were stained with anti-CD38-phyocerythrin and anti-CD45-allophycocyanin-Cy7 (BD Biosciences) to identify MM cells 62. All samples were collected following an Emory University Institutional Review Board (IRB)-approved protocol. CD138+ cells were purified using MACS Miltenyi Biotec CD138+ human microbeads as per instructions (#130-051-301). IC50s of venetoclax alone or in combination with TTFA/IACS in myeloma samples is calculated using non-linear regression (curve-fit) analysis under agonist vs normalized response with variable slope using GraphPad Prism 5.
Instrument	Samples were acquired on a FACSCanto II RUO Special Order System flow cytometer from BD (Becton Dickinson, San Jose, CA)
Software	De Novo Software FCS Express Version 4.
Cell population abundance	Primary multiple myeloma patient bone marrow aspirates containing a minimum of 5% CD138+ live cells in the isolated buffy coat were utilized for subsequent ex vivo studies.

Gating strategy

Cell lines analyses were on ungated cells. For patient samples we first gate on CD38+ CD45- cells followed by a live/dead analysis

☒ Tick this box to confirm that a figure exemplifying the gating strategy is provided in the Supplementary Information.
