# Supplementary material for: Electron transport chain activity is a predictor and target for venetoclax sensitivity in multiple myeloma
Source: Nat Commun. 2020 Mar 6;11:1228. doi: 10.1038/s41467-020-15051-z (PMC7060223; doi:10.1038/s41467-020-15051-z)
Supplement: Supplementary file 2 — Peer Review File [file 41467_2020_15051_MOESM2_ESM.pdf]

Reviewers' comments:

Reviewer #1 (Remarks to the Author); expert in Bcl2 inhibition:

Succinate ubiquinone reductase predicts and regulates venetoclax sensitivity in multiple myeloma

By Bajpai et al.

The authors investigated parameters of venetoclax sensitivity in multiple myeloma. They report that electron transport chain genes are suppressed in multiple myeloma and that myeloma cells that are sensitive to venetoclax exhibit reduced succinate ubiquinone reductase activity. They went on to show that SQR inhibition or mutant SQR enhances sensitivity to venetoclax.

Major points:

Figure 1:
To explore whether sensitivity to venetoclax is associated with reduced cellular energetics it is recommended to extend the analysis to additional cancer entities beyond multiple myeloma. Also, the study should be extended to non-malignant normal cells and their sensitivity to venetoclax to determine whether the correlation of venetoclax sensitivity and cellular energetics is specific for cancer cells or also applies to non-malignant cells.

Figure 2:
It is recommended to analyze expression levels of complex I and complex II to determine whether the activity levels of these complexes are associated with expression levels of key components of these complexes.

Figure 3:
In addition to pharmacological inhibitors of respiratory chain complexes, it is recommended to use genetic tools to block activity.

Figure 4:
Additional assays are required to determine whether SQR inhibition sensitizes multiple myeloma cells to venetoclax-mediated cell death in the long run. To this end, long-term assays, for example colony formation assays, should be used.

Figure 5:
To knock down one particular gene it is important to use at least two distinct siRNA constructs, since siRNA constructs might cause off-target effects.

Figure 7:
According to the model, ATF4 represents a critical link between the respiratory chain and proapoptotic BCL-2 family proteins. However, the current evidence supporting this critical role of ATF4 is still sparse. Therefore, additional experiments are required to determine whether or not ATF4 indeed exerts a pivotal role in the regulation of venetoclax sensitivity in multiple myeloma cells

by regulating BCL-2 family proteins.

Reviewer #2 (Remarks to the Author); expert in metabolism:

This paper demonstrates that mitochondrial SQR activity inversely correlates with venetoclax sensitivity. The study is very well done and conclusions are bolstered by data. The findings will be of great interest clinically to the use of Venetoclax.

I am bit surprised that they did not link SQR activity to H2S.
Very little is known about H2S and cancer. They have an opportunity to make this link and should.

Sulfide (H2S) is an inhibitor of mitochondrial cytochrome oxidase comparable to cyanide. Sulfide quinone oxidoreductase (SQR) converts H2S to a persulfide and transfers electrons to coenzyme Q via a flavin cofactor. Thus, a decrease in SQR would increase H2S and cause cytochrome c oxidase inhibition. While increase in SQR activity would decrease H2S. See the work of Ruma Banerjee at University of Michigan.

Here is my suggestion and key experiments to test:

1. Do agents that generate H2S sensitize to ventoclax resistant cells which have high SQR cells?
2. Please test whether cytochrome c oxidase inhibitors like Azide and NO (DETA-NO) sensitizes ventoclax resistant cells SQR positive cells.
3. Does SQR cDNA decrease sensitivity of cells low in SQR activity?

Cytochrome inhibition would sensitize cells to apoptosis as it would facilitate the release of cytochrome c.

Reviewer #3 (Remarks to the Author; expert in multiple myeloma:

The authors main assertion upon concluding this paper is that' disruption of SQR activity is sufficient to induce bcl2 dependence'. Unfortunately based on the data presented I simply cannot agree with this conclusion. They initially show metabolic differences between 2 pairs of cell lines of differing venetoclax sensitivity which seems reasonably robust but of limited consequence.They then go on to inform the remainder of the study with an analysis of a public data-base of expression data from t(11;14) and non-t(11;14) patients (with unknown venetoclax sensitivity). The rational given, being that the presence or absence of this genetic lesion is a proxy for venetoclax sensitivity. This is clearly flawed as only a minority of of patients in either group will be sensitive to venetoclax so what they are detecting are differences between these genetic sub-groups and not differences between sub-groups with varying venetoclax sensitivity. Subsequently SQR inhibition is shown to sensitise some but not all resistant cell lines (even though they manifest basically the same biochemical sequelae to SQR inhibition) but more tellingly only induces venetoclax sensitivity in 33% of the primary tumours they assessed. There may well be a correlation between venetoclax sensitivity and SQR expression (with very limited clinical relevance based on the primary tumour data they present) but I believe the conclusions made by the authors far over-reach the supportive data they present.

We appreciate the thorough review of our manuscript and the constructive comments. We have included several additional results (4 new panels in Figure 5 and 6 new supplementary figures) and changes to the text which we believe will significantly strengthen our conclusions. We look forward to your review of this revised manuscript. Our response to the critique is presented below (in italics) and all major changes to the text highlighted in yellow in the manuscript.

Reviewer #1 (Remarks to the Author); expert in Bcl2 inhibition:

NCOMMS-19-05940

Succinate ubiquinone reductase predicts and regulates venetoclax sensitivity in multiple myeloma

By Bajpai et al.

The authors investigated parameters of venetoclax sensitivity in multiple myeloma. They report that electron transport chain genes are suppressed in multiple myeloma and that myeloma cells that are sensitive to venetoclax exhibit reduced succinate ubiquinone reductase activity. They went on to show that SQR inhibition or mutant SQR enhances sensitivity to venetoclax.

Major points:

Figure 1:

To explore whether sensitivity to venetoclax is associated with reduced cellular energetics it is recommended to extend the analysis to additional cancer entities beyond multiple myeloma. Also, the study should be extended to non-malignant normal cells and their sensitivity to venetoclax to determine whether the correlation of venetoclax sensitivity and cellular energetics is specific for cancer cells or also applies to non-malignant cells.

This is a relevant suggestion and we assessed whether reduced SQR activity correlates with venetoclax sensitivity in other cancers (DLBCL, CLL and renal cancer lines).

We tested 6 DLBCL (SUDHL-4, SUDHL-6, U2932, OCI-LY19, OCI-LY10, HBL-1) lines that are known (and we confirmed) to show a range of sensitivity to venetoclax. We found that SQR activity did not correlate with venetoclax sensitivity. Results are included in Supplementary

Figure S6 a,b. Similar results were also obtained in a limited number of CLL patient samples. We also tested two renal cell cancer (HLRCC) cell lines, UOK268 (expressing mutant FH protein) and UOK269 expressing the SDHBR46Q mutation in SDHB which results in reduced SDHB expression. We anticipated both these mutants to have an accumulation of succinate and low SQR activity. Both these lines were however not sensitive to single agent venetoclax or sensitized to venetoclax with TTFA treatment. Normal, untransformed mature B cells are in fact known to be sensitive to venetoclax as reported in "*Leukemia (2014) 28, 1207–1215 Khaw, SL et.al: Title: Both leukaemic and normal peripheral B lymphoid cells are highly sensitive to the selective pharmacological inhibition of prosurvival Bcl-2 with ABT-199"*. We isolated normal B lymphocytes from peripheral blood and found them to have low SQR activity correlating with their venetoclax sensitivity. On the other hand, normal CD38 negative cells contained within the treated myeloma bone marrow aspirate were not sensitive to the TTFA + venetoclax co-treatment (as shown in Supplementary Figure S11). We summarized these observations in the text (page 9) and in the discussion (pages 16-17).

In sum, it appears that low Complex II (SQR activity) or Complex II inhibition may specifically increase BCL-2 dependence in the context of a plasma cell background, with our rationale below that is included in the discussion pages 16-17: "The correlation between low SQR activity and venetoclax sensitivity was not detected in other cancers such as in DLBCL cell lines (and in a preliminary evaluation of a small number of CLL patient samples and renal cancer lines). Interestingly normal, untransformed mature B cells are known to be sensitive to venetoclax {Khaw, 2014 #1348}. We isolated normal human B lymphocytes from peripheral blood and found them to have low SQR activity and sensitivity to venetoclax (data not shown). The activation/ differentiation of normal murine B cells is associated with increasing ABT-737 (a Bcl-2, Bcl-X_L, and Bcl-W antagonist) sensitivity associated with a decline in BCL-2 and increase in BCL-xL expression upon differentiation to a plasma cell {Gaudette, 2014 #1350}. Additionally, prior studies testing the effects of ABT-737 on the murine immune system showed ABT-737 to inhibit the establishment of newly arising bone marrow plasma cells with no effect on germinal center B cells {Carrington, 2010 #1351}. These results suggest that in B cells, BCL-2 dependence increases in an activation and/or differentiation-stage specific manner and declines upon differentiation of the B cell to a plasma cell. With respect to metabolism, B cell activation and differentiation to a plasma cell is associated with an increased dependency on the TCA and OXPHOS {Waters, 2018 #1352}{Price,

2018 #1358}. These results suggest that BCL-2 dependence appears to inversely correlate with a differentiation dependent elevation in OXPHOS.

t(11;14) MM are differentiated antibody producing plasma cells however early studies examining expression profiles of CD138+ MM in the CD2 subgroup of CCND1/CCND2-amplified MM cells identified elevated expression of B cell lineage markers like MS4A1/CD20, VPREB and PAX5 {Zhan, 2006 #286}. Thus a multiple myeloma (plasma cell malignancy) which should be exhibiting elevated OXPHOS may get locked into metabolically compromised state upon expressing/maintaining B cell like features leading to suppression of the ETC and SQR activity. Interestingly the non t(11;14) OCI-MY5 line that exhibits low SQR activity and venetoclax sensitivity is also likely to be more B cell-like or undifferentiated based on its high expression of CD20 (VA Gupta and LH Boise, unpublished observations). In sum, the B cell like features promoting BCL-2 dependence might also prevent the activation of OXPHOS repressing ETC/SQR activity in a cell that should normally be reliant on elevated OXPHOS. This suppression of the ETC activity is thus also associated with the sensitivity to venetoclax, allowing for a potent strategy of synthetic lethality to venetoclax. These studies have allowed us to also unravel an ETC target i.e. SQR to increase sensitization to venetoclax in ETC proficient plasma cells."

Figure 2:

It is recommended to analyze expression levels of complex I and complex II to determine whether the activity levels of these complexes are associated with expression levels of key components of these complexes.

We have now included expression levels of Complex I subunit: NADH dehydrogenase [ubiquinone] iron-sulfur protein 2/ NADH-ubiquinone oxidoreductase (NDUFS2) across the sensitive and resistant lines as described in results section page 9 and included in Supplementary Figure S5A. Complex II subunits in sensitive *vs* resistant cells were previously included (currently Supplementary Figure S5B). For the evaluated proteins in both Complex I and Complex II, expression levels do not correlate with their activity. For e.g. U266, KMS18 and RPMI-8226 are venetoclax-resistant cells but have low SDHA levels. In addition, most resistant cell lines have relatively higher SDHC expression (except KMS18 and RPMI-8226).

Likewise Complex I activity levels were lower in sensitive cells and do not correspond with expression of the Complex I subunit NDUFS2. It appears that the activity of these complexes do not entirely correlate with protein expression of the tested subunits as evident from both protein (New Supplementary Figure 5a,b) and RNASeq analysis (Fig 1I).

Figure 3:

In addition to pharmacological inhibitors of respiratory chain complexes, it is recommended to use genetic tools to block activity.

We have now tested the effects of Complex I suppression by knocking down (KD) NDUFS2. This 49 kDa protein is a core subunit of the mitochondrial membrane respiratory chain NADH dehydrogenase (Complex I). Concordant with piericidin not sensitizing resistant cells to venetoclax, NDUFS2 KD with individual siRNAs did not sensitize L363 and KMS11 lines to venetoclax. These results i.e. KD efficiency and sensitivity to venetoclax are described in the results section page 9 and now included in Supplementary Figure S9.

We have previously demonstrated the ability of the SDHC-R72C mutant to sensitize resistant multiple myeloma (MM) to venetoclax. SDHC KO cells did not survive in culture that lead to our strategy of knocking out the SDHC subunit and re-introducing SDHC or the SDHC-R72 mutant into resistant lines. This genetic perturbation of SDH subunit C activity sensitized resistant cells to venetoclax similar to pharmacological targeting with the Complex II inhibitor TTFA.

Figure 4:

Additional assays are required to determine whether SQR inhibition sensitizes multiple myeloma cells to venetoclax-mediated cell death in the long run. To this end, long-term assays, for example colony formation assays, should be used.

We have now performed colony forming assays in two myeloma cell lines (L363 and KMS11). Results of these assays are described in the results section page 10 and data included in Supplementary Figure S7. The combination of TTFA with venetoclax was in fact even more effective at sensitization than the single agents at lower doses in the longer duration 3D colony forming assays.

Figure 5:

To knock down one particular gene it is important to use at least two distinct siRNA constructs, since siRNA constructs might cause off-target effects.

We have now used the de-convoluted ATF4 SiRNA pool. We tested the effects of the individual siRNAs in two myeloma cell lines (KMS11 and JJN3). The individual SiRNAs demonstrate variable ATF4 KD efficiency and confirm our previous results obtained with the

ATF4-targeting SiRNA smartpool demonstrating a requirement for ATF4 induction in the TTFA induced-sensitization of MM to venetoclax. This new data is now described in the results section (page 11) and included in Supplementary Figure S10.

Figure 7:

According to the model, ATF4 represents a critical link between the respiratory chain and proapoptotic BCL-2 family proteins. However, the current evidence supporting this critical role of ATF4 is still sparse. Therefore, additional experiments are required to determine whether or not ATF4 indeed exerts a pivotal role in the regulation of venetoclax sensitivity in multiple myeloma cells by regulating BCL-2 family proteins.

This is an important point. So far our data demonstrates that 1) SQR inhibition induces ATF4; 2) ATF4 suppression reverses sensitivity to venetoclax; 3) ATF4 induction correlates with an induction of BIM and NOXA (known targets of ATF4) that are well characterized to elevate BCL-2 dependence as reported in (Morales AA*, et al.* and L.H Boise: Distribution of Bim determines Mcl-1 dependence or codependence with Bcl-xL/Bcl-2 in Mcl-1-expressing myeloma cells. *Blood* **118**, 1329-1339, 2011) and *Yuxuan Liu et.al and Anas Younes: NOXA genetic amplification or pharmacologic induction primes lymphoma cells to BCL2 inhibitor-induced cell death, PNAS, 2018)*; 4) SQR inhibition/TTFA treatment increases BIM binding to BCL-2 as demonstrated in a co-ip of BCL-2 and evaluation of BIM bound to BCl-2; 5) ATF4 KD or BIM KO suppresses venetoclax sensitivity. Additionally, we have now used NOXA knock out MM cell lines and demonstrate a loss of TTFA induced sensitization to venetoclax. We also show induction of ATF4 in these TTFA treated NOXA KO cells. 3 new panels related to the use of the NOXA KOS are presented in Figure 5. These results in sum, more comprehensively establish that the induction of ATF4 is pivotal for the induction of NOXA and BIM that in turn are necessary for TTFA induced sensitization to venetoclax. We have included these new results on page 12 of the text and in Figure 5 panels l,m,n,o and expanded our explanation for how ATF4 induction is pivotal to inducing BCL-2 dependence in myeloma cells.

Reviewer #2 (Remarks to the Author); expert in metabolism:

This paper demonstrates that mitochondrial SQR activity inversely correlates with venetoclax sensitivity. The study is very well done and conclusions are bolstered by data. The findings will be of great interest clinically to the use of Venetoclax.

I am bit surprised that they did not link SQR activity to H2S.

Very little is known about H2S and cancer. They have an opportunity to make this link and should.

Sulfide (H2S) is an inhibitor of mitochondrial cytochrome oxidase comparable to cyanide. Sulfide quinone oxidoreductase (SQR) converts H2S to a persulfide and transfers electrons to coenzyme Q via a flavin cofactor. Thus, a decrease in SQR would increase H2S and cause cytochrome c oxidase inhibition. While increase in SQR activity would decrease H2S. See the work of Ruma Banerjee at University of Michigan.

Here is my suggestion and key experiments to test:

1. *Do agents that generate H2S sensitize to ventoclax resistant cells which have high SQR cells?*

This is an excellent line of reasoning and we investigated this biology as outlined below. For clarity we are referring to the sulfide quinone oxidoreductase activity as (SQRDL) to distinguish it from the succinate ubiquinone reductase activity (SQR) focus of our paper. We treated venetoclax resistant MM lines with a dose range of sodium hydro sulfide (NaHS) which should be converted to the persulfide to inhibit Complex IV and potentially cause sensitization. NaHS (dose range upto 10mM and varying durations of treament) did not sensitize cells to venetoclax. To further understand why the exogenously added NaHS did not sensitize MM to venetoclax we investigated SQRDL expression in MM lines from the online myeloma Keats cell line repository (https://myelomagenomics.tgen.org/results_v3.php). Importantly, myeloma cells (both venetoclax sensitive and resistant) have barely detectable levels of SQRDL expression (< 0.5 FPKM) which would suggest the inability to convert H2S to the persulfide. This could be a possible reason for why NaHS did not induce sensitivity to venetoclax in the resistant MM lines.

Additionally, we tried treatment of venetclax sensitive MM cells with a persulfide scavenger (hydroxocobalamin) which should theoretically reverse any Complex IV suppression by accumulated H2S (due to lack of SQRDL expression). Hydroxocobalamin however had no

impact on venetoclax sensitivity further supporting H2S may not be accumulated in sufficient quantities to inhibit Complex IV in single-agent venetoclax sensitive cells. Thus H2S synthesis and potential for inhibition of Complex IV while relevant and interesting may not be at play in myeloma.

2. *Please test whether cytochrome c oxidase inhibitors like Azide and NO (DETA-NO) sensitizes ventoclax resistant cells SQR positive cells.*

We first tested whether Complex IV inhibition (with low doses of sodium azide) could sensitize MM to venetoclax. Indeed, MM cells were sensitized to venetoclax with sodium azide which supports a previous study where the oncometabolite (R)-2-HG expressed in IDH 1/2 mutant-expressing acute myeloid leukemia (AML) induces BCL-2 dependence and venetoclax sensitivity by suppressing cytochrome C oxidase (Complex IV) Chan SM*, et al.* Isocitrate dehydrogenase 1 and 2 mutations induce BCL-2 dependence in acute myeloid leukemia. *Nat Med* **21**, 178-184 (2015). We have cited this reference and our observation on the sodium azide effects on sensitizing MM to venetoclax in the discussion (page 19)

3. Does SQR cDNA decrease sensitivity of cells low in SQR activity?

This is a good point. We did overexpress SQR cDNA i.e. SDH C in addition to other SDH subunits in the low SQR activity expressing cells in order to reverse sensitivity to Ven. This strategy however did not lead to a reversal of sensitivity to Ven. We have noted in our results and discussion that Complex II activity levels are not completely dictated by expression levels which may explain the reason for lack of reversal of Ven sensitivity upon overexpression of SDHC.

Reviewer #3 (Remarks to the Author; expert in multiple myeloma:

The authors main assertion upon concluding this paper is that' disruption of SQR activity is sufficient to induce bcl2 dependence'. Unfortunately based on the data presented I simply cannot agree with this conclusion. They initially show metabolic differences between 2 pairs of cell lines of differing venetoclax sensitivity which seems reasonably robust but of limited consequence. They then go on to inform the remainder of the study with an analysis of a public data-base of expression data from t(11;14) and non-t(11;14) patients (with unknown venetoclax sensitivity). The rational given, being that the presence or absence of this genetic lesion is a proxy for venetoclax sensitivity. This is clearly flawed as only a minority of of patients in either group will be sensitive to venetoclax so what they are detecting are

differences between these genetic sub-groups and not differences between sub-groups with varying venetoclax sensitivity. Subsequently SQR inhibition is shown to sensitize some but not all resistant cell lines (even though they manifest basically the same biochemical sequelae to SQR inhibition) but more tellingly only induces venetoclax sensitivity in 33% of the primary tumours they assessed. There may well be a correlation between venetoclax sensitivity and SQR expression (with very limited clinical relevance based on the primary tumour data they present) but I believe the conclusions made by the authors far over-reach the supportive data they present.

We appreciate the rigorous critique in relation to the translational relevance of our observations to myeloma therapy. Our main assertion that "*disruption of SQR activity is sufficient to induce bcl2 dependence"* stems from the introduction of the SDHCR72 mutant into resistant cells that lead to significant sensitivity to venetoclax. While this addresses a "sufficiency question" in the context of the tested cell lines, we agree that 2 (U266 and KMS21BM) of the 8 myeloma cell lines were resistant to pharmacological inhibition of SQR and sensitization to venetoclax. Our explanation for their resistance is as follows: KMS21BM and U266 line exhibit high basal, maximal and spare respiration and in contrast to the TTFA sensitized KMS11 cells, do not exhibit a reduction in spare respiratory capacity as described in results page 12 and data now included in Supplementary Figure 12.

The KMS21BM and U266 cells also did not induce the distal effectors ATF4, BIM or NOXA with TTFA treatment, that we have shown to be required for venetoclax induced cell death. For the patient samples treated with TTFA we have reanalyzed this data to segregate the extent of sensitization of patient MM cells to venetoclax with TTFA co-treatment. Combination of TTFA and venetoclax induces significantly more cell death than TTFA or venetoclax alone (Figure 6a). As this expert reviewer correctly points out only ~32% of samples are sensitized using a 100 nM venetoclax cutoff of sensitivity. However, we would note that 46/50 (92%) samples showed a reduced IC50 when treated with TTFA and venetoclax as opposed to just venetoclax and the IC50 was reduced by greater than half in 31/50 (62%) samples (Figure 6b). These data are now described in the results section page 13-14 and data included in Figure 6 (a,b), which we believe is a better representation of the data and demonstrates the sensitization effects of TTFA on venetoclax efficacy in the patient samples.

The isolated patient samples above, in contrast to cell lines, are not proliferating *ex vivo*. It is possible that proliferating cells (more characteristic of poor prognosis MM) are more dependent upon OXPHOS due to energetic and biosynthetic requirements of cell division and thus could make them more sensitive to targeting SQR activity. The basis for resistance in the smaller subset of cell lines and patient samples could be due to compensatory Complex I activity (leading to maintenance of ETC activity) sustained by NADH generated through elevated pyruvate carboxylase activity, glutamine anapleurotic metabolism or fatty acid

oxidation. It is also likely that other non-metabolic mechanisms influence BCL2-dependence, and as such it is expected that targeting SQR activity will not result in venetoclax sensitivity in all types of myeloma. These limitations are now further discussed (pages19). We have included the aforementioned section in the discussion.

Regarding our gene expression analysis of primary multiple myeloma samples from the CoMMpass trial, we agree that having matching venetoclax sensitivity data would be ideal. Given that this information is not available, we have used t(11;14) as a proxy as ~40% of t(11;14) patients are venetoclax sensitive whereas <6% of non-t(4;14) are venetoclax sensitive (Kumar et al. Blood 2017 130:2401-2409, Matulis SM, et al. Leukemia 2019 33(5):1291-1296). As this reviewer correctly notes there are limitations to this analysis. It does, however, provide confirmatory correlative, which was informed from our work studying the metabolism of venetoclax sensitivity in multiple cell lines. This data was further confirmed using 50 patient samples treated *ex vivo*. We now discuss the limitations of the CoMMpass gene expression analysis in the second paragraph of the discussion (pages 15-16) and point out that it will be important to couple gene expression data with venetoclax-sensitivity in future studies.

Reviewers' comments:

Reviewer #2 (Remarks to the Author):

i am satisfied.

Reviewer #4 (Remarks to the Author); to comment on original Reviewer #3, expert in multiple myeloma:

Reviewer 3's original concerns were around the author's findings that inhibiting SQR activity is sufficient to induce BCL2 dependence and Venetoclax sensitivity. As only some t(11;14) patients are sensitive to Venetoclax, Reviewer 3 was concerned that these finding were only applicable to genetic 'sub-groups' and not wholistic differences between Venetoclax sensitive vs. resistant subgroups (as 33% of primary tumors had sensitivity induced with SQR inhibition).

The authors address reviewer 3's concern in the following ways: 1) Authors originally stated that 30% of patient sensitivity to Venetoclax was determined by the cutoff of IC50 <0.1uM. This being a strict cut-off, they showcase that 46 of 50 (92%) were overall sensitized post combination with TTFA, with 62% of them showing >50% decrease in IC50. 2) That more b-cell feature the MM clone or population is, the more dependent on BLC2, and that t(11;14) patients express a number of B-cell lineage markers - thus suggesting a genetic explanation to Venetoclax sensitivity in this sub-population (of which is speculation and not determined experimentally).

Firstly, The authors showcase that low SQR activity showcases sensitivity to Venetoclax independent of the t(11;14) translocation, which may be an important and novel clinical biomarker for initial Venetoclax sensitivity. Their new additions address the concerns to Reviewer 3 by showcasing that 92% of all patient samples were sensitized to Venetoclax in some degree. However, the authors indicate in their manuscript edits, that while all 10 resistant patients had high SQR activity, only 4 resistant patients of 10 became sensitized with TTFA treatment. While the cell line data is very convincing, the heterogeneity within patient samples enables a number of t(11;14) patients to be still resistant to Venetoclax in the presence of an SQR inhibitor, and may be a flawed hypothesis in the resistant setting. Due to the previously reported synergistic effects of Venetoclax and MCL2 antagonists, it can be predicted that any mechanism that perturbs the equilibrium of anti-apoptotic proteins toward BCL2 dependencies (eg. displacement of MCL2 by NOXA as shown in this study) will enable sensitivity to Venetoclax.

The addition of a SQR inhibitor appears to sensitize all MM cell lines and most (92%) primary samples to Venetoclax. Whether this sensitization is at therapeutically relevantly levels is yet to be determined. However, the data herin will be great importance to the field as we better understand genetic stratification or biomarkers for Venetoclax trials, and provide useful mechanistic data for general MM sensitive to Venetoclax and the t(11;14) subtype.

Reviewer #5 (Remarks to the Author); to comment on original Reviewer #1, expert in Bcl2:

Bajpai et al. propose that by promoting respiration, succinate ubiquinone reductase (SQR) suppresses ATF4 activity. According to their model, if SQR is inhibited in certain types of myeloma cells, ATF4 rises and drives expression of the BH3-only protein NOXA. NOXA displaces BIM, which can inhibit MCL1, Bclxl and Bcl2, so that there is less Bcl2, and as a consequence, less Venetoclax is needed to cause the cells to undergo apoptosis.

Consistent with this model, the authors provide evidence that in the relevant cells ATF4 and Bim are necessary for SQR inhibition to increase Venetoclax sensitivity.

Put another way, these experiments showed that in some myeloma lines, inhibition of mitochondrial respiration can promote activation of the Bax/Bak1 cell death mechanism, so that less Venetoclax is require to trigger apoptosis.

The experiments are well performed, scientifically sound, and clearly described. I have no scientific criticisms, but I do wonder about novelty and whether they be of interest to others in the community and the wider field:

1. How general these finding are: the authors found that inhibition of SQR only sensitises a subset of myeloma lines to Venetoclax, and when asked by a previous reviewer to look at other cell types, they found in DLBCL lines, CLL patients samples, and renal cancer lines, SQR activity did not correlate with Venetoclax sensitivity.

2. How novel these findings are: many drugs and toxins (including those that inhibit mitochondrial respiration) have been shown to induce Bax/Bak1 dependent apoptosis as a stress response.

3. How useful these findings will be: predicting myeloma sensitivity based on presence of the t(11;14) translocation is likely to be of more practical use than measuring SQR activity. As only a subset of t(11;14) myelomas respond well to Venetoclax as a single agent, I think more will be gained by testing drugs in combination with Venetoclax, rather than by trying to predict sensitivity based on SQR activity.

We appreciate the opportunity to have our manuscript reviewed a second time. As evident from the new referees' comments, those previous concerns appear to have been adequately addressed. The new referees however have some additional valid concerns that we strongly believe can be addressed. The original critique is pasted below. Our response is presented in italics.

Reviewer #4 (Remarks to the Author); to comment on original Reviewer #3, expert in multiple myeloma:

"Reviewer 3's original concerns were around the author's findings that inhibiting SQR activity is sufficient to induce BCL2 dependence and Venetoclax sensitivity. As only some t(11;14) patients are sensitive to Venetoclax, Reviewer 3 was concerned that these finding were only applicable to genetic 'sub-groups' and not wholistic differences between Venetoclax sensitive vs. resistant subgroups (as 33% of primary tumors had sensitivity induced with SQR inhibition).

The authors address reviewer 3's concern in the following ways: 1) Authors originally stated that 30% of patient sensitivity to Venetoclax was determined by the cutoff of IC50 <0.1uM. This being a strict cut-off, they showcase that 46 of 50 (92%) were overall sensitized post combination with TTFA, with 62% of them showing >50% decrease in IC50. 2) That more b-cell feature the MM clone or population is, the more dependent on BLC2, and that t(11;14) patients express a number of B-cell lineage markers - thus suggesting a genetic explanation to Venetoclax sensitivity in this sub-population (of which is speculation and not determined experimentally).

Firstly, The authors showcase that low SQR activity showcases sensitivity to Venetoclax independent of the t(11;14) translocation, which may be an important and novel clinical biomarker for initial Venetoclax sensitivity. **Their new additions address the concerns to Reviewer 3 by showcasing that 92% of all patient samples were sensitized to Venetoclax in some degree.** However, the authors indicate in their manuscript edits, that while all 10 resistant patients had high SQR activity, only 4 resistant patients of 10 became sensitized with TTFA treatment. While the cell line data is very convincing, the heterogeneity within patient samples enables a number of t(11;14) patients to be still resistant to Venetoclax in the presence of an SQR inhibitor, and may be a flawed hypothesis in the resistant setting. Due to the previously reported synergistic effects of Venetoclax and MCL2 antagonists, it can be predicted that any mechanism that perturbs the equilibrium of anti-apoptotic proteins
toward BCL2 dependencies (eg. displacement of MCL2 by NOXA as shown in this study) will enable sensitivity to Venetoclax.

The addition of a SQR inhibitor appears to sensitize all MM cell lines and most (92%) primary samples to Venetoclax. Whether this sensitization is at therapeutically relevantly levels is yet to be determined. However, the data herin will be great importance to the field as we better understand genetic stratification or biomarkers for Venetoclax trials, and provide useful mechanistic data for general MM sensitive to Venetoclax and the t(11;14) subtype"

We thank you for the careful review and critique. Our response to the highlighted portion of this critique is presented below in italics. We strongly believe the additional concerns can be adequately addressed:

This is in part due to:

1) a manuscript editing error on our part where we failed to update the new patient sample sensitivity analysis. In the updated analysis ***7*** *out of 10 resistant patient samples exhibited > 50% reduction in venetoclax IC50 upon co-treatment with TTFA. We had described the new analysis in the results section but failed to update this in the Figure 6 (Panel e) and in the discussion section of the resubmission (Page 14). This has now been updated in the text on page 14 and Figure 6 has also been revised. We have also uploaded Supplementary Table 1.*

2) our new finding of a translationally relevant electron transport chain (ETC) Complex I inhibitor that sensitizes resistant myeloma to venetoclax. The original reviewer #2 had asked whether other ETC Complex inhibition sensitizes multiple myeloma (MM) to venetoclax. This spurred our investigation of the other ETC Complexes.

We found that in addition to inhibition of Complex II, inhibition of Complex I, III, IV and V sensitize MM to venetoclax, directly answering Reviewer #2s question. These observations have not been comprehensively reported previously. Additionally, our new results support the combinatorial application of the newly developed Complex I inhibitor IACS-010759 with venetoclax.

To clarify further, during the process of investigating the other ETC complexes we found that the dose of piericidin (Complex I inhibitor) we used in the initial submission was 1 nM (not 1 μM) and that doses of piericidin 5 nM and above did sensitize myeloma cells to venetoclax (this data is now included in the supplement). We therefore tested the newly developed Complex I inhibitor IACS-010759 (in Phase I clinical trials for other cancers), and found that similar to piericidin, IACS-010759 sensitizes MM to venetoclax. Our new findings now enable us to directly address the therapeutic significance of inhibiting the ETC to improve sensitivity to venetoclax in MM patients. We have ex vivo patient sample data demonstrating that both t(11;14) and non t(11;14) patient samples are sensitized to venetoclax with IACS-010759 co-treatment. We have included this new data in an updated Figure 7. We believe these additional observations make our study highly translational as there is still a need to elicit deeper responses with less toxicity in MM.

We agree it is well known that perturbing the binding partners of the pro-apoptotic proteins can shift BCL-2 or MCL-1 dependence. Our study builds on this known concept to identify mitochondrial targets to elevate BCL-2 dependence and increase the primed state of BCL-2 by increasing BIM binding to BCL-2. This elevation in BCL-2 dependence is driven in part by the induction of NOXA that binds to MCL-1 to shift BIM to BCL-2. This strategy saturates MCL-1 to preclude re-sequestration of BIM. Currently the strategies in clinical trial to increase venetoclax sensitivity in MM rely on the addition of dexamethasone with or without bortezomib and venetoclax in t(11;14) patients, a strategy that is showing some relapse and toxicity. In ex vivo studies, while dexamethasone was found to sensitize the majority of resistant t(11;14) and non t(11;14) patient samples to venetoclax, a subset were completely refractory to the combination (Matulis, SM, Leukemia. (2019) 33:1291-1296), warranting the continued identification of new methods to sensitize resistant MM to venetoclax. The lack of sufficient priming of a cancer cell in general, is linked to resistance to a number of therapeutics warranting the need to identify means of bringing a cancer cell closer to the apoptotic threshold further underscoring the need to identify new strategies to capitalize on the use of BCL-2 antagonists like venetoclax*. Now, utilizing a Complex I inhibitor (IACS-010759) currently in Phase I trials, and therefore of translational therapeutic significance, we demonstrate that Complex I inhibition also sensitizes myeloma to venetoclax. This new data is presented in revised Figure 7. As the reviewer correctly points out, previous reports (such as from our co-author Dr Boise, L (Nature Communications **9**: 5341 2018) and others Raje, N (Leukemia. 2019 (8):2098-2102) and the Ocio, E (Haematologica July 2019) groups have demonstrated the additive/synergistic effects of venetoclax and MCL-1 antagonists ex vivo. The mechanistic basis for this synergy essentially relies on the release of sufficient BIM above the apoptotic threshold by targeting two BCL-2 family members. The dual application of an MCL-1 and BCL-2 antagonist however has not moved into clinical trials and in fact the Phase I trial testing the single agent MCL-1 inhibitor AMG 397 and AMG 176 have been placed on clinical hold due to indications of cardiac toxicity. Previous studies suggest MCL-1 may be required for normal cardiac myocyte mitochondrial activity/survival further supporting the continued identification of new targets to elevate BCL-2 dependence without directly targeting MCL-1.*

Minimal residual disease (MRD) in MM is connected to the emergence of refractory disease. Therefore, it is beneficial to identify new strategies that will elicit deeper responses upfront. Targeting Complex II for venetoclax sensitivity has in fact been described in AML using azacytidine in a series of papers from the Jordan group. Our study, however, uniquely parses out how it is sufficient to target the SQR activity of Complex II or Complex I to achieve potent sensitization to venetoclax. The compound we used to target SQR is a tool compound, that sensitized 92% of the patients samples we tested, and sets the premise for the development of more potent small molecule inhibitors targeting SQR.

Reviewer #5 (Remarks to the Author); to comment on original Reviewer #1, expert in Bcl2:

“Bajpai et al. propose that by promoting respiration, succinate ubiquinone reductase (SQR) suppresses ATF4

activity. According to their model, if SQR is inhibited in certain types of myeloma cells, ATF4 rises and drives expression of the BH3-only protein NOXA. NOXA displaces BIM, which can inhibit MCL1, Bclxl and Bcl2, so that there is less Bcl2, and as a consequence, less Venetoclax is needed to cause the cells to undergo apoptosis.

Consistent with this model, the authors provide evidence that in the relevant cells ATF4 and Bim are necessary for SQR inhibition to increase Venetoclax sensitivity.

Put another way, these experiments showed that in some myeloma lines, inhibition of mitochondrial respiration can promote activation of the Bax/Bak1 cell death mechanism, so that less Venetoclax is require to trigger apoptosis.

The experiments are well performed, scientifically sound, and clearly described. I have no scientific criticisms, but I do wonder about novelty and whether they be of interest to others in the community and the wider field:

1. How general these finding are: the authors found that inhibition of SQR only sensitizes a subset of myeloma lines to Venetoclax, and when asked by a previous reviewer to look at other cell types, they found in DLBCL lines, CLL patients samples, and renal cancer lines, SQR activity did not correlate with Venetoclax sensitivity."

CLL samples are already highly sensitive to single venetoclax that is related to elevated BCL-2 expression. B cell malignancies arising from different stages of B cell development have discrete biology that may be related to their elevated BCL-2 dependence. We believe identifying that low ETC activity/SQR activity correlates with increased venetoclax sensitivity in the second most common hematological malignancy i.e. multiple myeloma is an important finding that can be capitalized upon for functional biomarker-based profiling in the future.

2. "How novel these findings are: many drugs and toxins (including those that inhibit mitochondrial respiration) have been shown to induce Bax/Bak1 dependent apoptosis as a stress response."

Currently the strategies in clinical trial to increase venetoclax sensitivity in MM rely on the addition of dexamethasone with or without bortezomib and venetoclax in t(11;14) patients that is showing some relapse and toxicity. In ex vivo studies while dexamethasone was found to sensitize the majority of resistant t(11;14) and non t(11;14) patient samples to venetoclax, a subset were completely refractory to the combination (Matulis, SM, Leukemia. (2019) 33:1291-1296), warranting the continued identification of new methods to sensitize resistant MM to venetoclax. Now, utilizing a Complex I inhibitor (IACS-010759) currently in Phase I trials, we have outlined a therapeutically relevant strategy to increase venetoclax sensitivity in both t(11;14) and non t(11;14) patients that could lead to deeper upfront responses. The lack of sufficient priming of a cancer cell in general is linked to resistance to a number of therapeutics warranting the need to identify means of bringing a cancer cell closer to the apoptotic threshold further underscoring the need to identify new strategies to capitalize on the use of BCL-2 antagonists like venetoclax. *Our new data related to this translationally relevant Complex I inhibitor is included in Figure 7.*

Minimal residual disease (MRD) in MM is connected to the emergence of refractory disease. Therefore, it is beneficial to identify new strategies that will elicit deeper responses upfront. Targeting Complex II for venetoclax sensitivity has in fact been described in AML using azacytidine in a series of papers from the Jordan group. Our study however uniquely parses out how it is sufficient to target the SQR activity of Complex II or Complex I to achieve potent sensitization to venetoclax. The compound we used to target SQR is a tool compound, that sensitized 92% of the patients samples we tested, and sets the premise for the development of more potent small molecule inhibitors targeting SQR.

3. "How useful these findings will be: predicting myeloma sensitivity based on presence of the t(11;14) translocation is likely to be of more practical use than measuring SQR activity. As only a subset of t(11;14)

myelomas respond well to Venetoclax as a single agent, I think more will be gained by testing drugs in combination with Venetoclax, rather than by trying to predict sensitivity based on SQR activity."

Our results lay the foundation for the development of mitochondrial functional readouts to predict sensitivity. We have found a strong positive correlation between SQR activity and venetoclax sensitivity. Further, low SQR activity in 4 patient samples, including two non-t(11;14) corresponded with ex vivo venetoclax sensitivity suggesting that SQR activity is a reliable predictor of venetoclax sensitivity irrespective of the t(11;14) status. We are focusing on furthering this interrogation and believe that our report will also spur greater interest in the development of such assays as functional biomarkers. We also agree that there is a need for more agents to increase venetoclax sensitivity. We thus now provide results on the efficacy of a translationally relevant small molecule currently in clinical trials i.e. IACS-010759. In fact, this drug sensitizes non t(11;14) MM and a t(11;14) patient sample that are unresponsive to venetoclax treatment ex vivo, suggesting the utility of testing this treatment strategy in MM.

REVIEWERS' COMMENTS:

Reviewer #2 (Remarks to the Author):

I am satisfied.

Reviewer #4 (Remarks to the Author):

Thank you to the authors for addressing the previous concerns.

Our main concern was around the only 4/10 patients becoming sensitized to Venetoclax treatment with TTFA treatment. However, the re-analysis and update of 7/10 patients confirms significance in clinical settings.

Their claims are further supported with clinical and novel Complex I inhibitors (IACS-010759).

Therefore, I support publication/acceptance of this manuscript to this journal.

Reviewer #5 (Remarks to the Author):

This paper is scientifically sound and the authors' conclusions are supported by the data presented. The only criticisms I have relate to novelty and practical impact.
